# Cystic liver lesions: a pictorial review

**DOI:** 10.1186/s13244-022-01242-3

**Published:** 2022-07-15

**Authors:** Mégane Chenin, Anita Paisant, Jérôme Lebigot, Paul Bazeries, Kawtar Debbi, Maxime Ronot, Valérie Laurent, Christophe Aubé

**Affiliations:** 1 Department of Radiology, University Hospital of Angers, 4 rue Larrey, 49 933 Angers Cedex 09, France; 2 HIFIH Laboratory, EA 3859, UNIV Angers, 49045 Angers, France; 3 Department of Radiology, La Timone Children Hospital of Marseille, 264 rue St Pierre, 13385 Marseille Cedex 05, France; 4 Department of Radiology, Beaujon Hospital, APHP.Nord, 100 boulevard du Général Leclerc, 92110 Clichy, France; 5 Department of Radiology, University Hospital of Nancy, 29 avenue du Maréchal de Lattre de Tassigny, 54035 Nancy, France; 6 Université de Paris, Paris, France

**Keywords:** Hepatic, Cysts, Ultrasonography, Computed tomography, Magnetic resonance imaging

## Abstract

Hepatic cysts (HC) are sac-like structures mainly filled with liquid and showing a distinct membrane. They are usually found incidentally through imaging. A wide spectrum of imaging patterns may be observed for common and uncommon, neoplastic and non-neoplastic diseases. While simple hepatic cysts occur frequently and do not require any treatment or follow-up, non-typical cysts should be carefully analysed to avoid misdiagnosing a lesion that would require appropriate management. Therefore, adequate knowledge of all the relevant imaging patterns is critical to secure an accurate diagnosis. The aim of this review is to describe the imaging features of the different types of hepatic cysts.

## Key points

- Cystic liver lesions are common lesions dominated by simple hepatic cysts.
- Aetiologies of hepatic cysts are varied: developmental, infectious, neoplastic.
- Atypical characteristics of hepatic cysts should always be searched for.
- Atypical features are: thick wall, septa or solid portion, enhancement, non-fluid signal.
- The clinical context must always be considered.

## Background

Hepatic cysts are frequent (2.5% to 18% in the general population), mostly incidental and asymptomatic lesions [1–3]. They are well-defined fluid-filled lesions, with or without epithelial lining. Depending on the lesion, the cystic component may be composed of different types of fluid, including bilious, serous, mucinous, necrotic, haemorrhagic, proteinaceous or mixed fluid [4, 5]. Some of them may be diagnosed non-invasively on the basis of specific imaging features [5]. Radiologists should both know and recognise non-typical benign features—i.e. wall thickness, presence of septa, tissular component, enhancement, and signal intensity on MRI [7, 9]—and systematically take the clinical context into consideration.

The aim of this article is to describe and illustrate some of the most frequent cystic hepatic lesions, with a special emphasis on typical radiological features, which allow a non-invasive diagnosis. Even though the extra-hepatic bile ducts abnormalities, rare tumours sometimes cystic (hepatocellular carcinoma, mucinous cholangiocarcinoma, etc.) and post-traumatic lesions will not be addressed in this review, they are listed in Table 1.

**Table 1:** Other cystic lesions that could be evocated as differential diagnosis

Post-traumatic lesions (biloma, hematoma, seroma)
Pseudocyst following acute pancreatitis
Epidermoid cyst
Endometrial cyst
Cystic hepatocellular carcinoma
Undifferentiated embryonal sarcoma
Mucinous cholangiocarcinoma
Mesenchymal Hamartoma

**Fig. 1:**
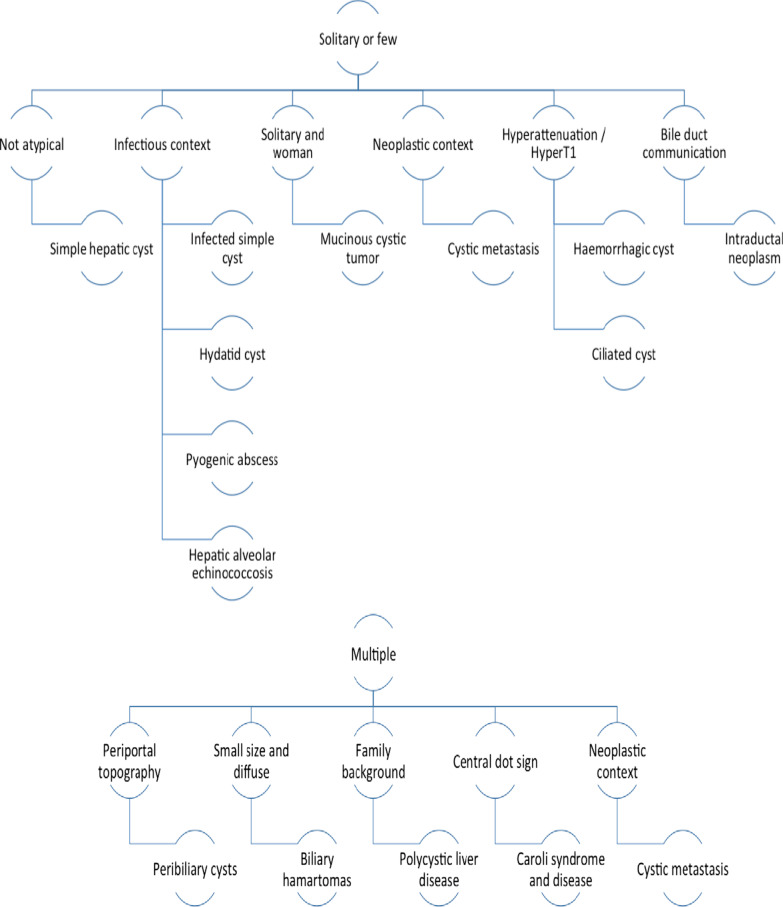
Simple algorithms helping to delineate diagnosis according to the number of cysts

A summary of the imaging patterns for each cystic disease is presented in Table 2. Finally, Fig. 1 shows a diagnostic algorithm.

**Table 2:** Summary of the imaging patterns for each cystic disease

Disease	US patterns	CT patterns	MRI patterns
Simple hepatic cyst	Anechoic Homogeneous Smooth margins Thin wall No mural nodule nor septation	Hypoattenuation near water Smooth margins Thin wall No mural nodule nor septation No enhancement	HypoT1, hyperT2 Smooth margins Thin wall No mural nodule nor septation No enhancement
Haemorrhagic cyst	Anechoic/hyperechoic Heterogeneous Non-enhancing septas “Fern leaf” aspect Possibly painful during US exam	Higher attenuation than water Hyperattenuation Fluid–fluid level No enhancement	Blood signal according to the stage of the bleeding Heterogeneous No enhancement
Infected cyst	Anechoic/hyperechoic Thick wall	Higher attenuation than water Thick enhancing wall Fluid–fluid level Gas bubbles	Heterogeneous intensity Thick enhancing wall Possibly restricted water diffusion (when large)
Bile duct hamartoma	Small and multiple Hyperechoic Comet-tail artefacts “Snow storm” aspect	Small and multiple Hypoattenuation Irregular shape No enhancement	Small and multiple HypoT1, hyperT2 “Starry sky” aspect No communication with the biliary tract
Caroli syndrome	Diffuse fusiform dilatation of the biliary tract “Central dot” sign Hepatic dysmorphia	Same as US Better visibility of the “central dot” sign Intrahepatic lithiasis	Same as CT Assessment of communication with the biliary tract
Caroli disease	Diffuse aneurysmal dilatation of the biliary tract “Central dot” sign	Same as US Better visibility of the “central dot” sign Intrahepatic lithiasis	Same as CT Assessment of communication with the biliary tract
Polycystic liver disease	Multiple simple hepatic cyst Compression	Multiple simple hepatic cyst Compression	Multiple simple hepatic cyst Compression
Peribiliary cyst	Multiple small peri-portal cysts “String of pearls” aspect	Multiple small peri-portal cysts “String of pearls” aspect	Not communicating with the biliary tract (MRCP and use of hepatobiliary MRI contrast agent may help)
Hepatic lymphatic malformation	Multilocular Enhancing thin septas on ECUS	Multilocular Enhancing thin septas and wall	Possibly varying signal on T1 and T2 Dilated lymph duct on lymphoMR
Ciliated hepatic foregut duplication cyst	Solitary and subcapsular Mostly in the IV segment Hypoechoic Possibly heterogeneous, fluid–fluid level	Solitary and subcapsular Mostly in the IV segment Hyperattenuation/hypoattenuation higher than simple fluid Fluid–fluid level No enhancement	Solitary and subcapsular Mostly in the IV segment HyperT2 and hyperT1 Fluid–fluid level No enhancement
Mucinous cystic neoplasm of the liver	Solitary and large Anechoic Multilocular Irregular margins Septas and thick wall	Solitary and large Hypoattenuation Multilocular Enhancing septas and (thick) wall Mural nodule (invasive type) Rare calcification (invasive type)	Solitary and large Varying signal on T1 and T2 Multilocular Enhancing septas and wall Fluid–fluid level/protein rich fluid Haemorrhagic fluid / mural nodule (invasive type)
Intraductal papillary neoplasm of the bile duct	Mostly intraluminal hypo or hyperechoic mass with upstream duct dilatation	Intraductal heterogeneous mass enhancing at arterial phase, non-increasing on portal and delayed phase	HyperT2 and hypoT1 heterogeneous Same enhancement as CT Connection with the biliary tract on MRCP Possibly restriction of diffusion
Cystic metastasis	Non-purely anechoic With thick wall, mural nodule, septas	Various type Hypoattenuation with complex patterns: thick wall, septas, enhancement, mural nodule	Various type HyperT2 with complex patterns: thick wall, septas, enhancement, mural nodule
Pyogenic abscess	Heterogeneous Anechoic/hypoechoic/hyperechoic	Hypoattenuation “Double target” sign “Honeycomb” pattern and “Cluster” sign Gas bubbles	HyperT2, hypoT1 Same patterns as CT Central restriction of diffusion (large abscess)
Hydatid cyst (first stages/patterns depend on the stage)	Anechoic with possibly mobile internal echos and hyperechoic parts Irregular margins	Hypoattenuation Irregular margins Wall calcifications Internal septas, thick wall	HyperT2 HypoT2 wall and septas Heterogeneous signal Typical “wheel-spoke” pattern for CE2
Hepatic alveolar echinococcosis	Hypoechoic/hyperechoic/anechoic Heterogeneous Irregular margins	Hypoattenuation Heterogeneous Irregular margins Central calcifications May deform the hepatic capsule No enhancement	HyperT2, hypo to intermediate T1 HypoT2 and hypoT1 for fibrotic part Multivesicular aspect

## Ductal abnormalities

**Fig. 2:**
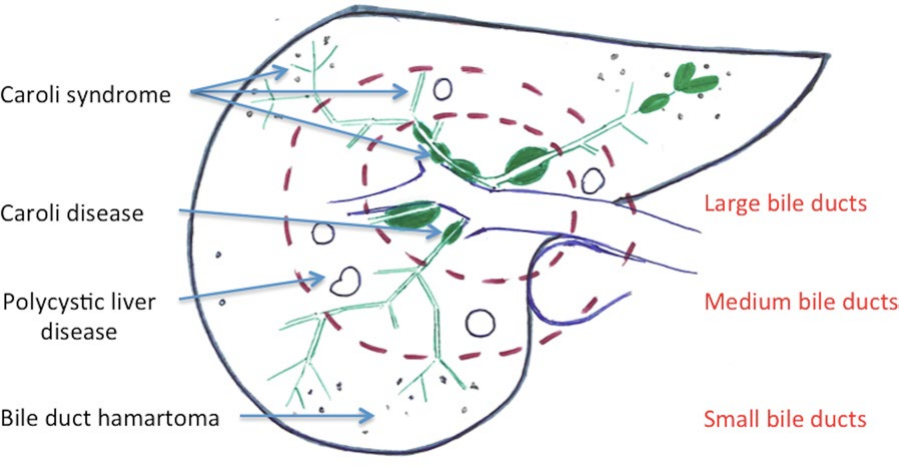
Schematic figure (axial section of a liver) showing the different types of ductal plate malformation. The size of the affected ducts is closely correlated to the ductal development phase

Ductal plate malformations correspond to a group of congenital cystic liver lesions, which can affect both the intra- and/or the extrahepatic biliary ducts. They result from insufficient remodelling and resorption of cylindrical ductal plates [8]. Figure 2 emphasises the stage of ductal plate malformation according to the development of the bile duct, from the centre to the periphery.

### Simple hepatic cyst

#### Aetiopathogenesis

Simple hepatic cysts (SHC) (also known as bile duct cysts or biliary cysts) are congenital parts of the ductal tree detached from the main biliary system, which dilated to become cystic lesion [9]. There is no consensus on their actual origin. They are thought by some authors to result from the dilatation of the bile duct hamartoma (cf. section) [4], and they do not communicate with the biliary tract. In the literature, their prevalence is reported to range from 2.5 to 18%, occurring more frequently in women, with an incidence that increases with age [5, 10, 11].

SHCs are benign lesions, which are usually asymptomatic. Cystic serous fluid is continually produced by the cuboidal biliary epithelium [10]. A very large cyst can cause abdominal pain or early satiety by compression. Complications, which may include haemorrhage or infection, are rare and may result in the lesion becoming a “complex cyst”.

#### Imaging

A SHC is a well-circumscribed unilocular lesion with a thin imperceptible wall, which may be lobulated; it is anechoic on ultrasonography (US) with increased through-transmission. A SHC is hypoattenuating on computed tomography (CT) images (0–20 HU) and appears as hypointense on T1 and strongly hyperintense on T2 images with magnetic resonance imaging (MRI). It does not have internal nodule and does not show enhancement after the administration of intra-venous contrast agents (whether with US, CT or MRI) (Figs. 3 and 4). It can be bi- or multi-lobar (fused cysts) (Fig. 6). The typical features on each imaging modality are sufficient findings allowing a confident diagnosis.

**Fig. 3:**
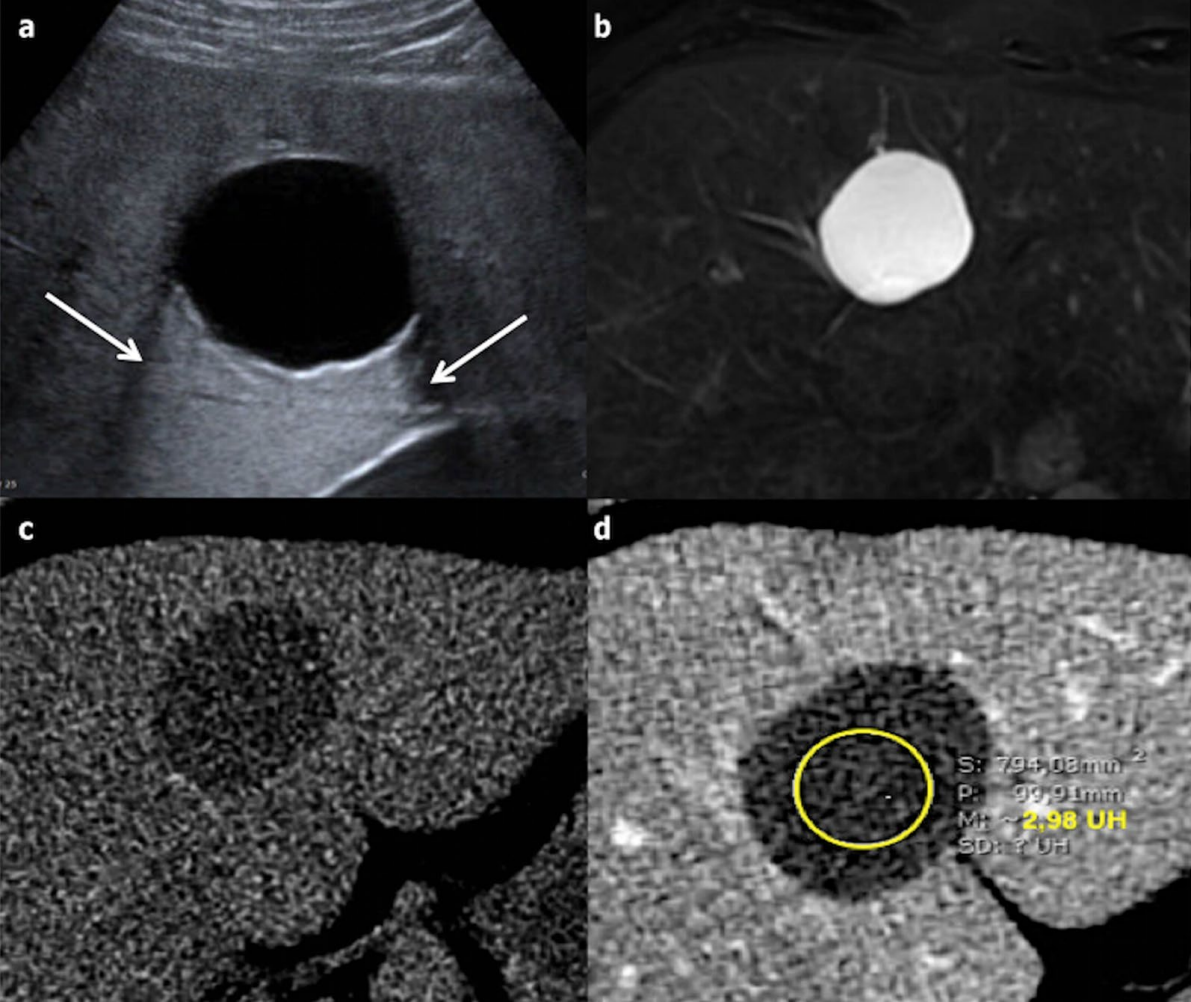
Incidentally found simple hepatic cyst in an asymptomatic 64-year-old male. **a** Ultrasonography shows a round homogeneous anechoic cystic hepatic lesion, well-circumscribed and without any mural nodule or vegetation. The arrows show an increased through transmission, confirming the cystic nature of the lesion. **b** T2-weighted magnetic resonance imaging shows a highly hyperintense round lesion. **c** and **d** Computed tomography without and with contrast on portal venous phase shows a round homogeneous non-enhancing hypoattenuating lesion (3 Hounsfield Units)

**Fig. 4:**
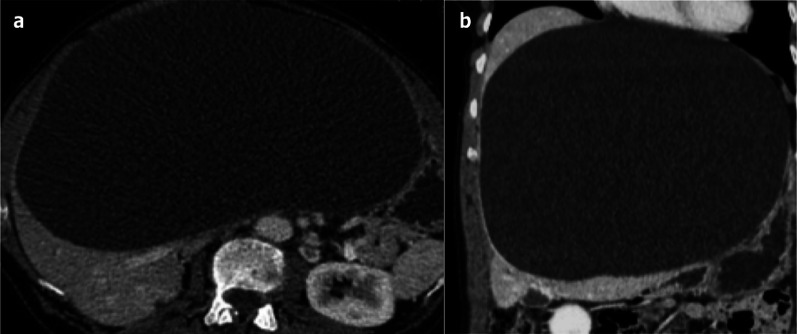
Very large simple hepatic cyst in a symptomatic 64-year-old female with abdominal pain. **a** Axial and (**b**) coronal computed tomography on portal venous phase shows a very large simple hepatic cyst. The cyst had grown progressively for a certain number of years and was eventually treated by laparoscopic fenestration, resulting in pain relief

#### “Complex cyst”

Complications of SHCs are rare. However, they need to be recognised, since they change the features of the cyst and could therefore lead to misdiagnosis. This is especially the case of tumoural cysts [11].

**Fig. 5:**
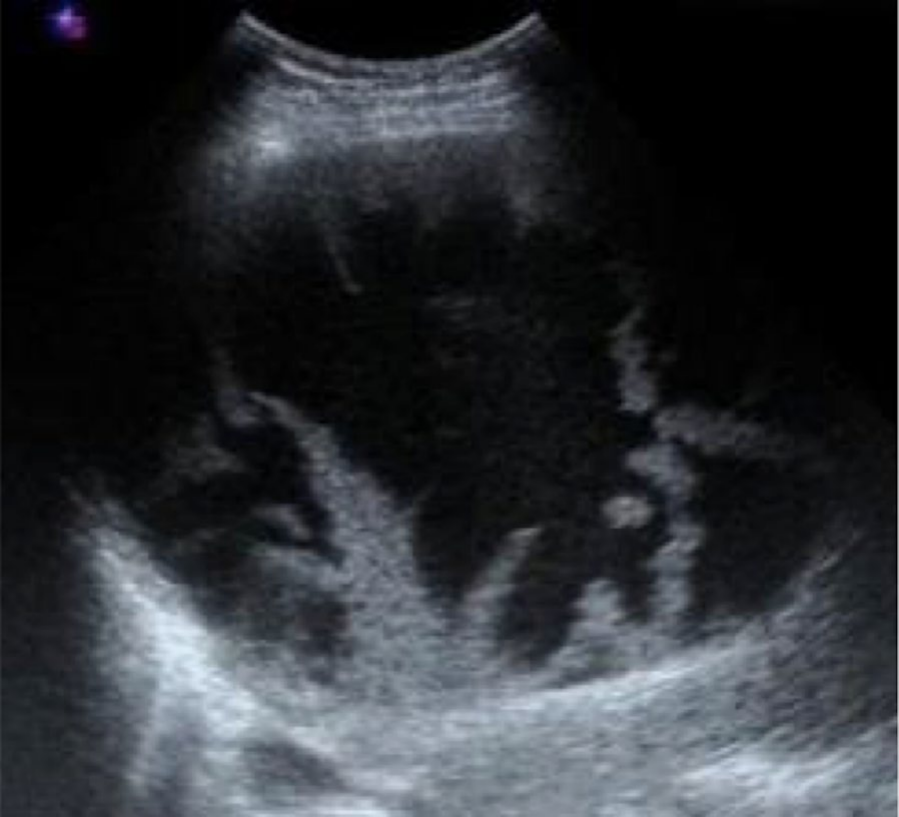
Subacute haemorrhage in a simple hepatic cyst in a 70-year-old male. Ultrasonography shows a spontaneous mobile area of hyperechogenicity inside the cyst, appearing as a “fern leaf”

**Fig. 6:**
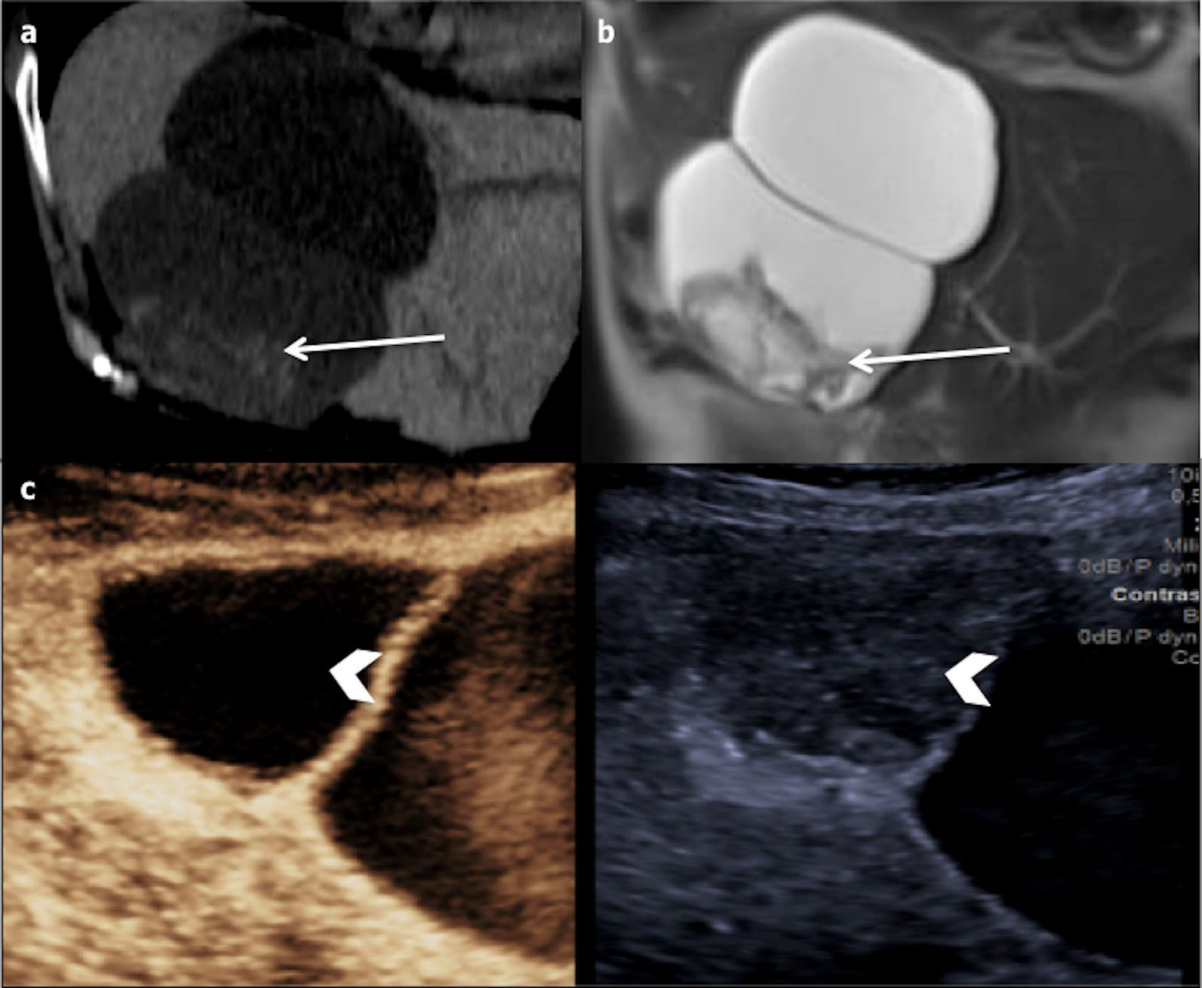
Bilobulated haemorrhagic hepatic cyst in a 73-year-old female. **a** Coronal non-enhanced computed tomography shows a bilobulated cyst with focal hyperattenuation in the inferior part (arrow); **b** coronal T2-weighted magnetic resonance imaging shows heterogeneous hypointensity inside the inferior part of the cyst (arrow). **c** Contrast-enhanced ultrasonography confirms the non-enhancement of the cystic part of the lesion (arrowhead)

**Fig. 7:**
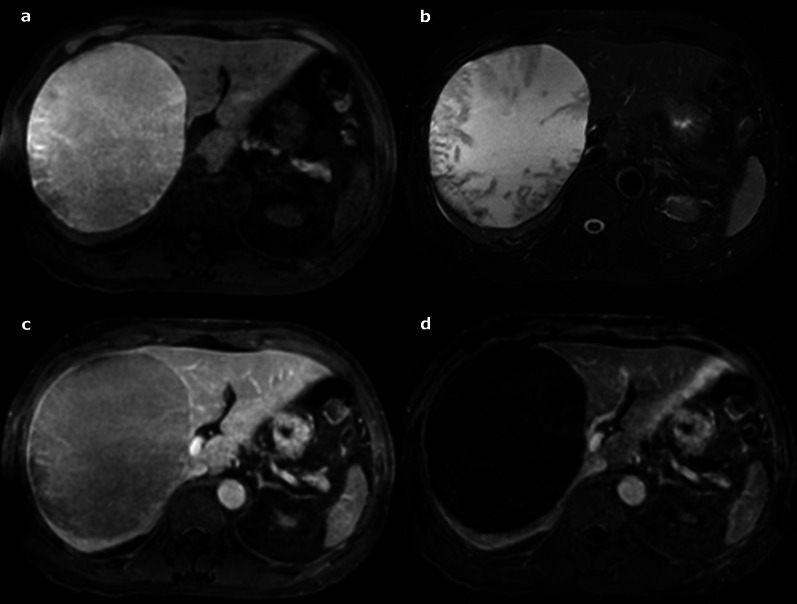
Haemorrhagic cyst in a 55-year-old male patient. **a** Axial T1 fat-sat-weighted magnetic resonance imaging shows hyperintense lesion and (**b**) axial T2-weighted magnetic resonance imaging shows a heterogeneous hyperintensity; **c** and **d** axial T1-fat-sat-weighted imaging without and with subtraction shows no enhancement after gadolinium-chelate injection

**Fig. 8:**
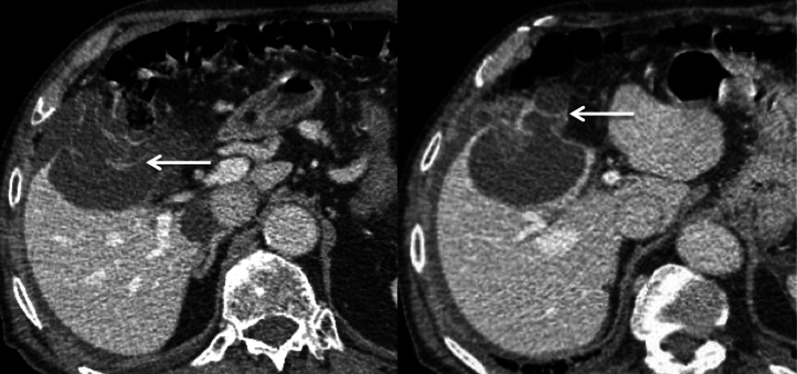
Rupture of a hepatic cyst in a 89-year-old male with severe abdominal pain. Axial computed tomography on portal venous phase demonstrates a floating wall (arrow) associated with pericystic and peri-hepatic fluid collection

- *Haemorrhage*: these cysts can increase in size. They have a heterogeneous content with septations seen as echoic intralesional materials (Fig. 5) (pain can occur during ultrasound examination). On CT, their attenuation is higher than that of simple fluid and, on MRI, the signal produced is that generated by blood flow according to the stage of the bleeding (Figs. 6 and 7). A fluid–fluid level can be observed (Fig. 6). The inner septa are mobile but do not enhance. A pseudo-capsule in the form of a thin peripheral rim enhancement is depicted and should not be mistaken for tumoural tissue.
- *Infections*: they are exceptional, except in the case of polycystic disease. A thick wall with heterogeneous enhancement, a fluid–fluid level and some gas bubbles inside the cyst can be seen. It can be difficult to differentiate these cysts from abscesses, unless a prior examination demonstrated the presence of SHCs.
- *Rupture*: rupture also occurs very rarely and a typical sign suggesting rupture is a “floating wall” inside the cyst. In case of a subcapsular cyst, a rupture can be associated with perihepatic fluid (Fig. 8).
- *Large symptomatic SHC*: even though very large SHCs are usually asymptomatic, they could cause pain or duodenogastric compression. The treatment may be surgical (cyst fenestration) or radiological by sclerotherapy. Due to the difficulty in attributing symptoms to the cyst, a diagnostic test can be carried out through a simple aspiration.

### Bile duct hamartoma

#### Aetiopathogenesis

Bile duct hamartomas (BH) (also known as von Meyenburg complexes) are benign lesions caused by failure of the small interlobular bile ducts to involute at the late phase of embryogenesis [12–14]. BHs are asymptomatic and disconnected bile duct lesions with only limited dilation, lined by biliary epithelium. The prevalence based on autopsy series is around 5.6% in adults [13]. BHs can be observed in healthy livers or in association with other ductal plate abnormalities [13]. As of 2016, 35 cases of cholangiocarcinoma associated with BH have been reported in the literature, without other associated ductal plate malformation [13].

#### Imaging

**Fig. 9:**
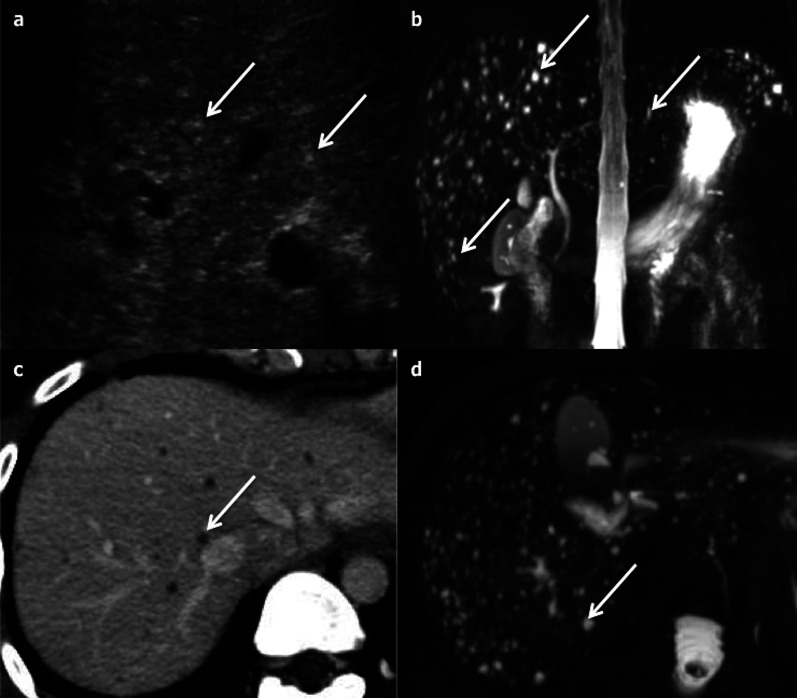
Bile duct hamartomas in a 52-year-old female. **a** Multiple hyperechoic round nodules (arrows) at ultrasonography, displaying a “snowstorm” pattern. **b** Coronal magnetic resonance cholangiopancreatography shows several small hyperintense nodules, which do not communicate with the biliary tract (arrows). **c** Axial computed tomography on portal venous phase shows bilobar small round-shaped hypoattenuating nodules, which can be better detected with an axial magnetic resonance cholangiopancreatography sequence (**d**)

BHs are commonly subcapsular or located at the periphery of portal tracts [12]. US images show multiple small (< 1.5 cm) intra-hepatic cysts, which are mostly hyperechoic due to their small size and to a fibrous stroma (Fig. 9a), with comet-tail artefacts [4, 13]. The pattern they produce has been described as a “snowstorm” [14]. They are poorly visible on CT, round or irregular in shape, with strict fluid attenuation and no enhancement (Fig. 9c). MRI allows a better detection with a strongly T2-weighted sequence [13] resulting in a “starry sky” appearance [15] (Fig. 9d). Magnetic resonance cholangiopancreatography (MRCP) sequences confirm the lack of communication with the biliary tract (Fig. 9b). In some cases, a thin regular persistent enhanced rim can be seen due to the compressed surrounding liver parenchyma [4]. The typical features on each imaging modality are sufficient to reach a conclusive diagnosis. It may be difficult to discriminate between bile duct hamartomas and simple hepatic cysts when there are only few bile duct hamartomas in the liver parenchyma. The main differentiating patterns include the size of the lesion (small) and their disposition (mostly peripheral). However, from a clinical standpoint, setting them apart is not critical, because both are benign lesions.

### Caroli syndrome and disease

These are both extremely rare congenital ductal plate development abnormalities (prevalence of less than one in 1,000,000 inhabitants) [16]. Caroli disease (CD) occurs less frequently than Caroli syndrome (CS). CS must be differentiated from CD. Their pathophysiology is different. CD results from the abnormal development of large bile ducts, whereas CS affects both the central and smaller peripheral bile ducts. CS combines cystic biliary dilatation (congenital intrahepatic ductal dilatation) and the features of congenital hepatic fibrosis, whereas CD is characterised solely by biliary dilatation. The origin of CD is unclear: it is thought to be usually nonhereditary, and it is rarely transmitted in an autosomal dominant mode. CS is transmitted in an autosomal recessive mode. While CD is assumed to result from a partial or complete interruption at an early phase of ductal plate remodelling, in CS, the abnormalities occur both at an early and at a late phase and involve peripheral bile ducts [8, 15]. Follow-up is mandatory for CS and CD due to the increased risk of cholangiocarcinoma [17]. In addition, the cysts and biliary tract can contain stones, resulting from the stagnation of bile.

#### Caroli disease

##### Aetiopathogenis

The lack of periportal biliary epithelium regression during embryogenesis creates the aneurysmal dilatation of the large intra-hepatic bile ducts [8, 15]. The liver parenchyma is normal. Frequent symptoms include abdominal pain, jaundice, and/or cholangitis.

##### Imaging

**Fig. 10:**
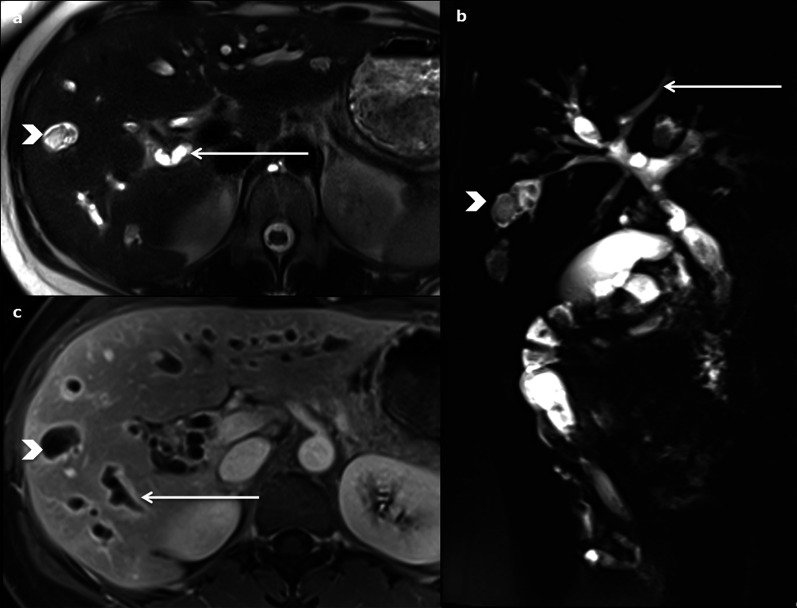
Caroli disease in a 24-year-old male. **a** Axial magnetic resonance imaging in T2 and (**b**) in T1 on portal venous phase show both fusiform (arrows) and connected round (arrowheads) dilatations, better depicted on magnetic resonance cholangiopancreatography images (**c**). There is no associated sign of fibrosis

The biliary tract is abnormal and there are focal dilated bile ducts, without stenosis (Fig. 10). The cystic dilatations are connected to the biliary tract and have thin walls with a “central dot sign,” corresponding to a residual portal vein and arterial branch [15]. The Doppler or contrast-enhanced CT/MRI images confirm its vascular origin [10].

MRCP is the modality of choice for assessing these lesions. Cystic hepatic lesions communicate with the biliary tract (Fig. 10). The use of hepatobiliary MRI contrast agents can be useful to confirm the communication between the biliary tracts and cysts [15]. There is no liver atrophy (Fig. 10).

#### Caroli syndrome

##### Aetiopathogenesis

This syndrome combines bile duct dilatations and congenital hepatic fibrosis. CS could be associated with the autosomal recessive polycystic kidney disease (PKHD1 mutation) [18]. The symptoms are due to the consequences of liver fibrosis. Follow-up is required not only to prevent the complications induced by hepatic fibrosis but also because of the increased risk of cholangiocarcinoma (about 7%) [17].

##### Imaging

**Fig. 11:**
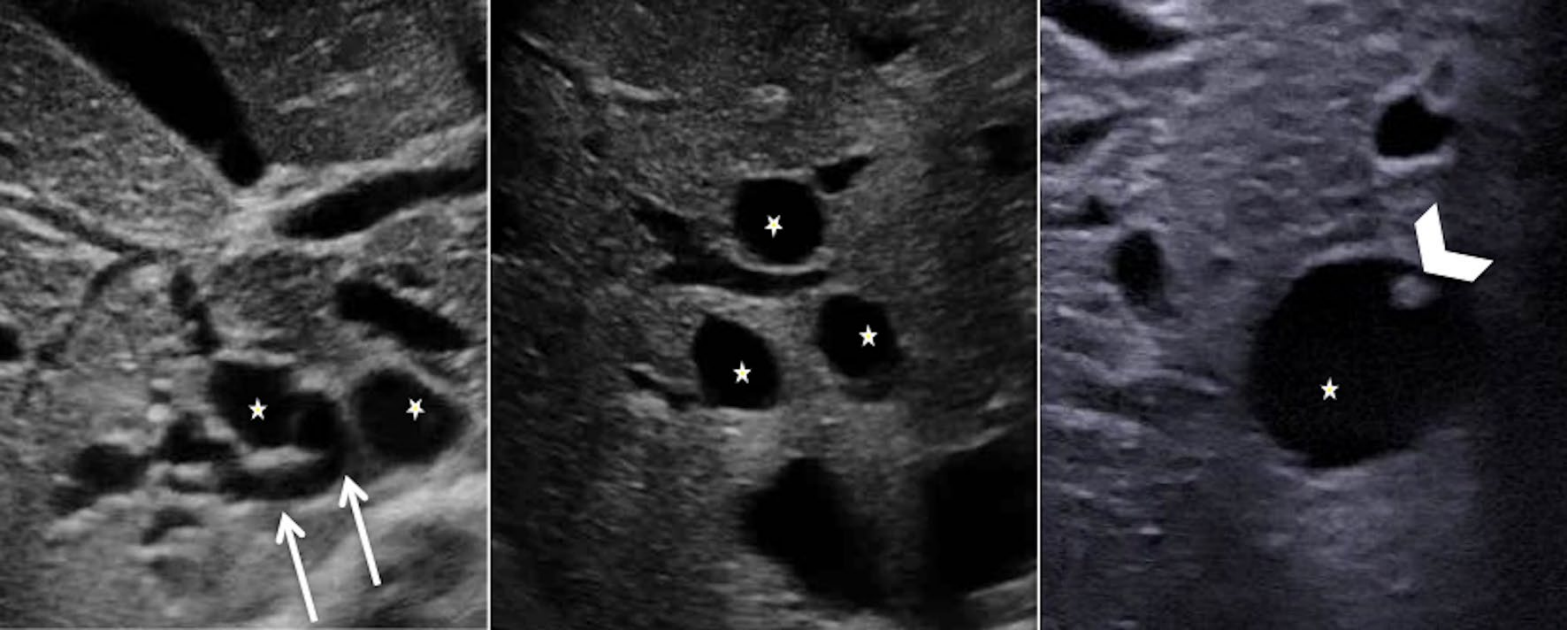
Caroli syndrome in a 7-year-old female, at ultrasonography. Several round anechoic cysts (stars) with thin wall, increased through-transmission, connected to the dilated fusiform biliary tract (arrows), some of which show the highly specific “central dot sign” (arrowhead)

The features are similar to those of CD, with cysts communicating with the dilated bile ducts, and with the specific “central dot sign” (Fig. 11). They are usually smaller in CS than in CD (< 3 cm), with diffuse fusiform dilatation of the biliary tract [10].

**Fig. 12:**
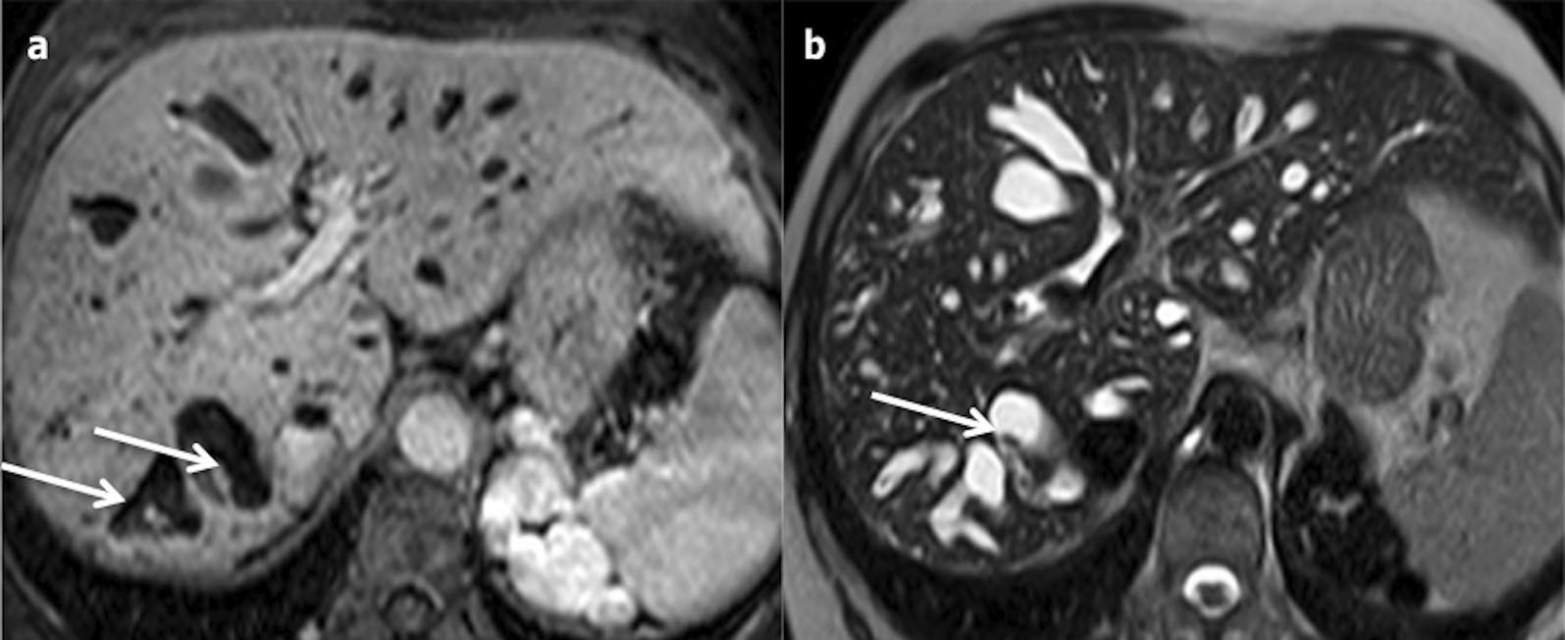
Caroli syndrome in a 53-year-old female. **a** Axial magnetic resonance imaging on portal venous phase shows peripheric fusiform dilated bile duct, hyperintense on axial T2-weighted magnetic resonance imaging (**b**), with the specific “central dot sign” (arrows). Note the presence of spleno-renal shunts due to portal hypertension

The associated hepatic fibrosis may lead to specific dysmorphia (hypertrophy of the left and caudate lobes, *normal size or hypertrophy* of segment IV and hypotrophy of the right lobe [19]) and to features of portal hypertension (portosystemic collateral vessels, splenomegaly and ascites) (Fig. 12).

### Polycystic liver disease

#### Aetiopathogenesis

**Fig. 13:**
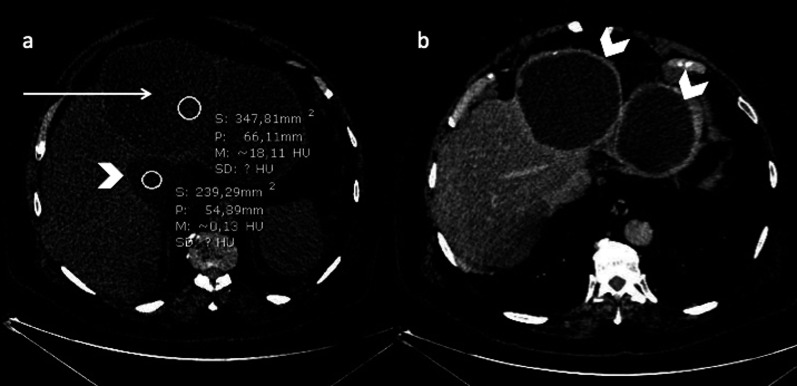
Infected simple hepatic cysts in a 77-year-old male with autosomal dominant polycystic liver disease. **a** Axial non-enhanced computed tomography shows two cysts under pressure (arrow), slightly more attenuating than the non-infected cysts (arrowhead); **b** with a thick enhanced wall on the portal venous phase (arrowhead)

**Fig. 14:**
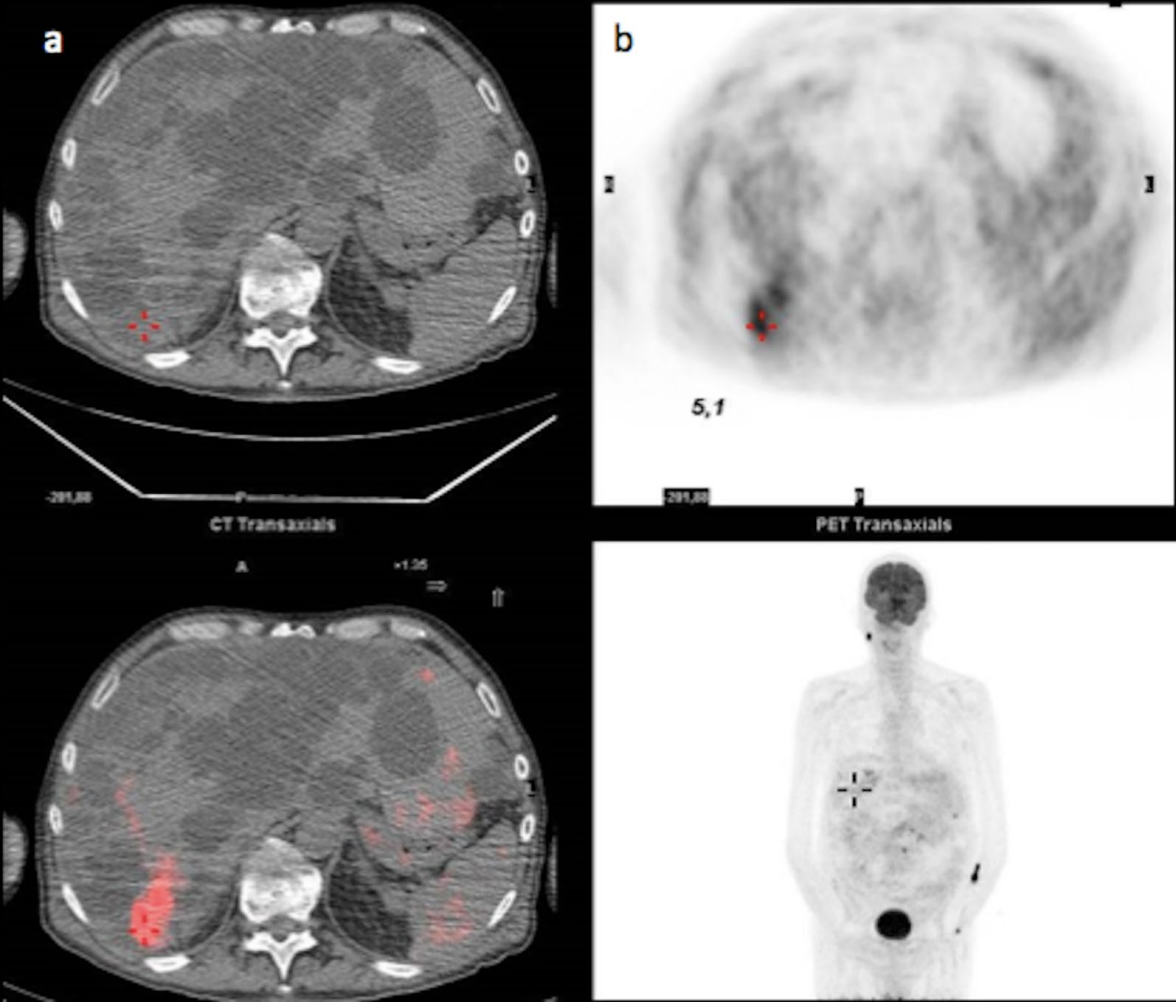
Infected cyst in a 65-year-old male patient with autosomal dominant polycystic liver disease. **a** Axial non-enhanced computed tomography depicts one more attenuating cyst among multiple simple cysts (cross); **b** 18-F-FDG PET imaging shows the increased metabolism of that cyst (SUV at 5,1)

Polycystic liver disease (PLD) may involve the liver only (autosomal dominant polycystic liver disease (ADPLD): ≈ 1:100,000), or the liver and the kidney, in a dominant (autosomal dominant polycystic kidney disease (ADPKD): ≈ 1:600–1,000) or recessive form (autosomal recessive polycystic kidney disease (ARPKD)) ≈ 1:20,000) [20]. The number of cysts required for diagnosis has not been clearly defined. The international PLD registry steering committee suggests that at least 10 simple hepatic cysts should be present for a diagnosis to be made [21]. As with SHCs, complications include organ compression (including biliary and hepatic vessels), haemorrhage, infection (Fig. 13) or rupture. The detection of an infected cyst among multiple ones might be difficult, but 18-Fluorine-fluorodeoxyglucose positron emission tomography (18-F-FDG PET) imaging may provide helpful guidance (Fig. 14).

#### Imaging

**Fig. 15:**
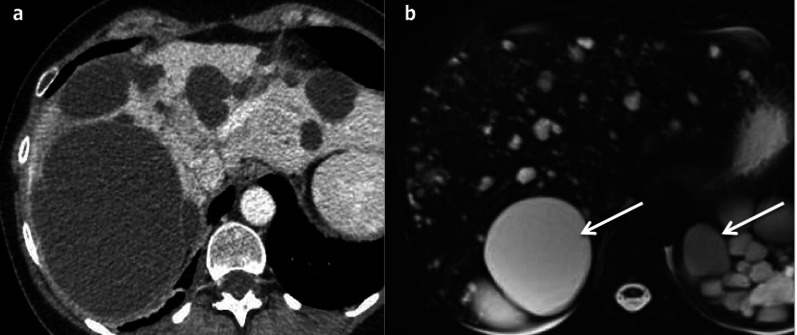
Autosomal dominant polycystic kidney disease in a 38-year-old male patient. **a** Axial computed tomography on portal venous phase and **b** axial T2-weighted magnetic resonance imaging show several simple hepatic cysts and also numerous kidney cysts (arrows), with fluid signal (hypodense/hyperintense on T2)

Cysts, which are similar to SHCs, are spread diffusely throughout the liver (Fig. 15). They may coalesce then showing a linear calcification between them. They frequently lead to hepatomegaly and to vascular compression with venous collaterals [22].

## Other cystic lesions

### Peribiliary cyst

#### Aetiopathogenesis

Peribiliary cysts, which are small serous cysts caused by obstructed extramural hilum peribiliary glands, must not be confused with biliary dilatations. The aetiopathogenesis of these cysts and their exact prevalence are unknown. They are mostly found in cirrhotic liver (50% in autopsy studies [23]), in ADPKD or ADPLD [24] and can be solitary (10%). They can sometimes cause compression and cholangitis. A classification of the cysts has been proposed according to their location: type I including intrahepatic biliary tract, type II extrahepatic biliary tract and type III intrahepatic and extrahepatic biliary tract (25).

#### Imaging

**Fig. 16:**
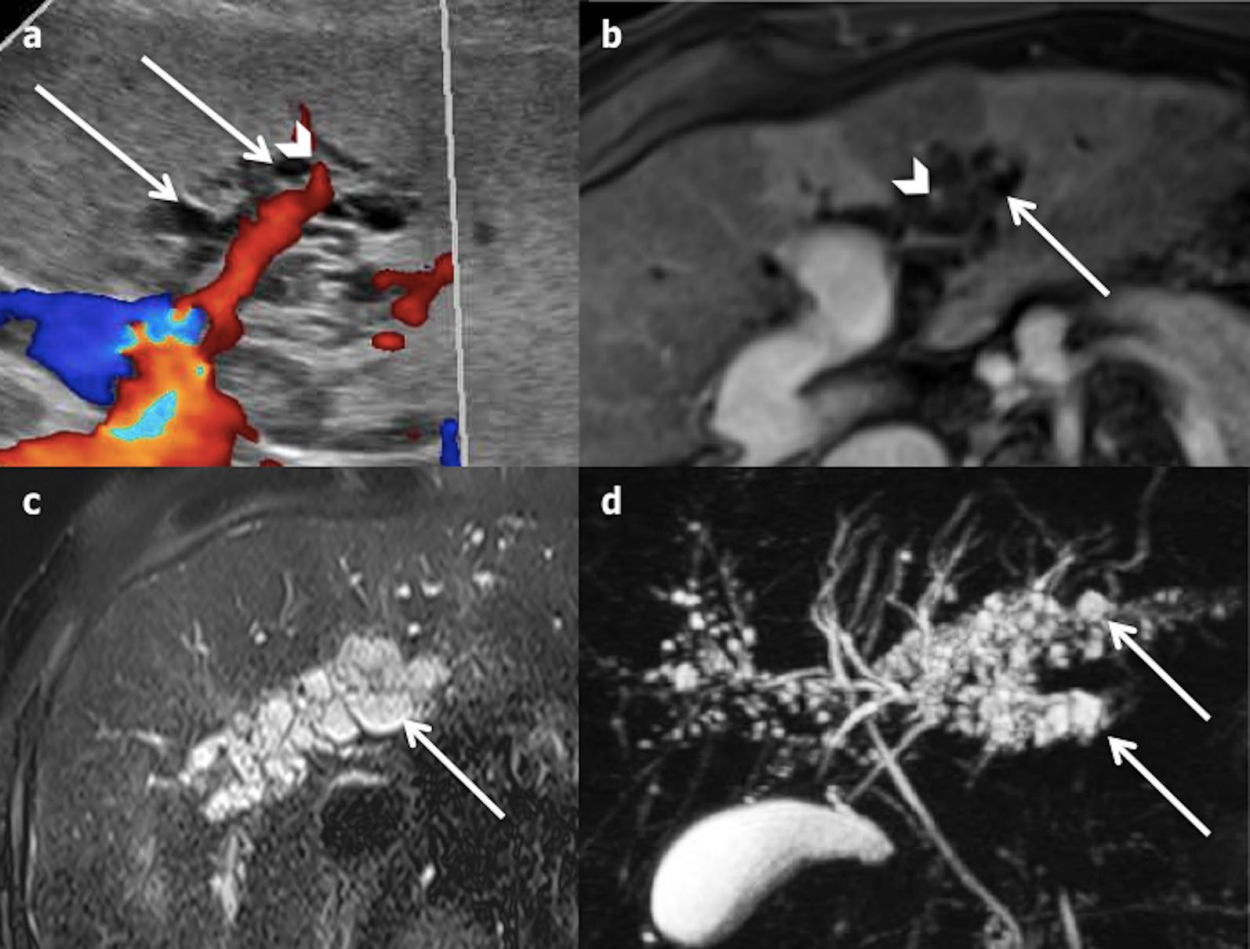
Peribiliary cyst in a 69-year-old cirrhotic male. **a** Ultrasonography shows multiple periportal (arrowhead) cysts (arrows). **b** The portal vessels are clearly visible (arrowhead) on axial portal venous phase magnetic resonance imaging, showing the periportal location of the cysts. **c** and **d** Axial T2-weighted magnetic resonance imaging and magnetic resonance cholangiopancreatography images show multiple small cysts along thin, non-dilated bile ducts

Peribiliary cysts are characterised by multiple, usually small (from 1 to 55 mm [24]), simple cystic lesions, appearing as a “string of pearls” around the hilar portal veins (Fig. 16). They are predominantly located in the left lobe due to the preponderance of the peribiliary gland [10]. While they do not communicate with the biliary tract, they can communicate with one another, thereby mimicking biliary dilatations. MRCP is helpful to differentiate them from biliary dilatations (Fig. 16) and hepatobiliary MRI contrast agent can be used to prove the absence of biliary communication [10]. On rare occasions, peribiliary cysts can be confused with dilated lymphatic vessels [25].

### Hepatic lymphatic malformation

#### Aetiopathogenesis

Hepatic lymphatic malformations (HLM) are extremely rare (8 cases reported in the literature up to 2010 [26]). They consist of benign cystic dilatations of the liver’s lymphatic spaces, commonly associated with systemic lymphangiomatosis [6, 27]. These cysts are lined with endothelial cells and filled with lymph [27]. Three subtypes of HLM are described in the literature: capillary (super-microcystic), cavernous (microcystic), or cystic (macrocystic) [6, 27]. Through immunohistochemistry, D2-40 has been reported to be a highly specific antibody for the identification of the lymphatic endothelium when making a diagnosis during histological analysis [

#### Imaging

**Fig. 17:**
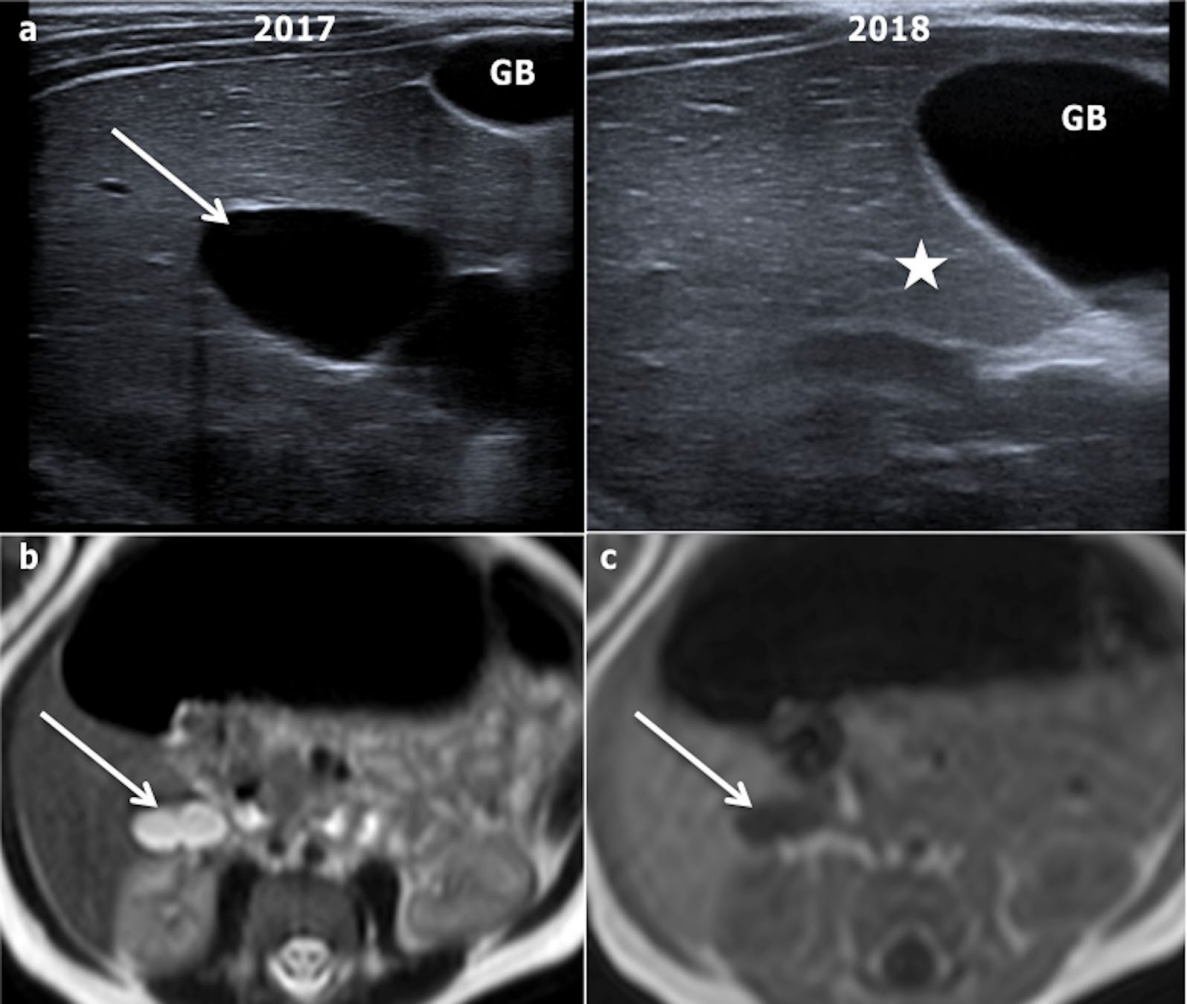
Spontaneous disappearance of a cystic lesion in a 5-year-old boy between 2017 and 2018, which led to the diagnosis of this lesion as a most likely liver lymphatic malformation. **a** Ultrasonography shows biloculated homogeneous cyst (arrow), which vanished one year later (star). It displayed fluid signal (arrows) on axial T2-weighted (**b**) and T1-weighted magnetic resonance imaging in 2017 (**c**). The main differential diagnosis of this lesion would be two fused simple hepatic cysts or mesenchymal hamartoma

They can be unilocular, but are more often multilocular, with septa and wall, which may enhance inconsistently. Confusion can occur with cystadenomas, cystadenocarcinomas or sclerosing haemangiomas [26, 29], but MRI can be usefully relied upon to avoid such a likelihood. The hepatic lymphatic malformations produce varying signals in T1- and T2-weighted sequences, depending on the quantity of fat and fluid components [30]. Lympho-MR sequences can show dilated lymph duct surrounding the lesion, thereby supporting the diagnosis of HLM [25, 28]. Finally, hepatic lymphatic malformations can spontaneously disappear (Fig. 17).

### Ciliated hepatic foregut duplication cyst

#### Aetiopathogenesis

Ciliated hepatic foregut duplication cyst (CHFC) is a very rare (about 100 cases reported in the literature up to 2012 [31]) and usually incidental finding. It seems to be caused by a congenital malformation from the embryonic foregut [31]. It comprises four layers: an inner pseudo-stratified columnar epithelial lining, a subepithelial loose connective tissue, smooth muscle and a fibrous capsule [32]. Abdominal discomfort may be due to its subcapsular localisation [33].

The diagnosis can only be made by histology or through fine needle aspiration showing ciliated pseudostratified tall columnar epithelial cells suspended in a mucoid background with immunohistochemical stains [34]. Complete surgical excision is recommended [32] due to the risk of malignancy, especially if the cyst is larger than 5 cm [35, 36].

#### Imaging

A small (< 4 cm) solitary subcapsular cyst in segment IV is very suggestive of CHFC. However, it has also been described in segment V or VIII [32, 33, 36]. Cysts are non-enhancing, without septa, partition or internal nodule.

**Fig. 18:**
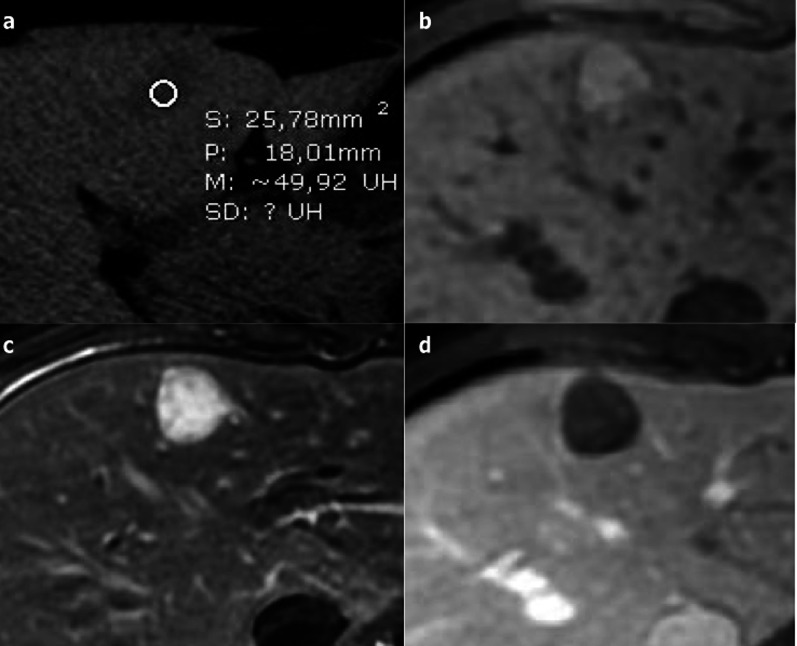
Ciliated hepatic foregut duplication cyst in a 15-year-old male. **a** Axial non-enhanced computed tomography shows a subcapsular nodule of the fourth segment with spontaneous attenuation value of around 50 Hounsfield Units. **b** On axial T1-weighted magnetic resonance imaging, it is spontaneously hyperintense. **c** It displays a high level of hyperintensity on axial T2-weighted magnetic resonance imaging. **d** Axial T1-weighted magnetic resonance imaging on portal venous phase shows neither contrast enhancement nor wall thickening

Its contents are usually fatty or protein-rich, which may lead to the creation of a fluid–fluid layer [36]. On US, CHFC are hypoechoic, but usually not completely anechoic; on CT, they can be spontaneously hyperattenuating. On MRI, cysts are hyperintense on T2-weighted images, but the signal intensity is nonetheless not as high as that of simple hepatic cysts. On T1 images, the signal is usually spontaneously hyperintense. CHFCs do not demonstrate any contrast enhancement (Fig. 18) [37].

However, classical signs can be missed when the component of the cyst varies.

The differential diagnosis includes: haemorrhagic cyst, mucinous cystic neoplasm of the liver, metastasis of melanoma, dysplastic nodule (on cirrhosis liver), epidermoid and endometrial cyst. Some imaging patterns can help to accurately identify the different lesions. The haemorrhagic cyst is frequently larger, more heterogeneous and may show a peripheral hypointense T2 wall. The mucinous cystic neoplasm may show enhanced inner septa and a thick enhanced wall. The metastases of melanoma are most of the time less hyperintense on T2 and may show a slight enhancement. The dysplastic nodule is not hyperintense on T2. The epidermoid cyst is very rare and may be differentiated by fine needle aspiration. The endometrial cyst usually has sub-capsular topography.

## Cystic tumours

### Mucinous cystic neoplasm of the liver

#### Aetiopathogenesis

Mucinous cystic neoplasms of the liver (MCN-L), previously known as biliary cystadenoma and cystadenocarcinoma, are rare cystic neoplasms (3–5% of hepatic cysts [9]) without biliary communication. MCN-L are composed of cuboidal to columnar epithelial cells, which variably produce mucin and which are associated with ovarian-type subepithelial stroma. They are subdivided into non-invasive and invasive types [38] and frequently occur in women. The diagnosis can be hinted at by the intralesional assay of specific tumour markers (TAG-72) [39].

Treatment requires surgery, since imaging cannot differentiate non-invasive from invasive types with certainty [9].

#### Imaging

They are frequently large (1.5–35 cm) solitary multilocular cystic lesions, mostly located close to the hepatic hilum in segment IV, with well-circumscribed irregular margins and septations. Internal septa can be calcified [3]. Septations arising from the internal cyst wall without external indentation seem to be a specific sign that can be relied upon to differentiate MCN-L from SHC [7].

On US, they appear as large unilocular anechoic lesions, with thickened and irregular walls and internal septations [40]. Contrast-enhanced ultrasonography (CEUS) can confirm the enhancement of inner septa.

On CT, they appear as a solitary hypoattenuating lesion with septations, a thick wall, rarely with calcification [40]. The attenuation of the cyst varies, depending on the nature of the component.

**Fig. 19:**
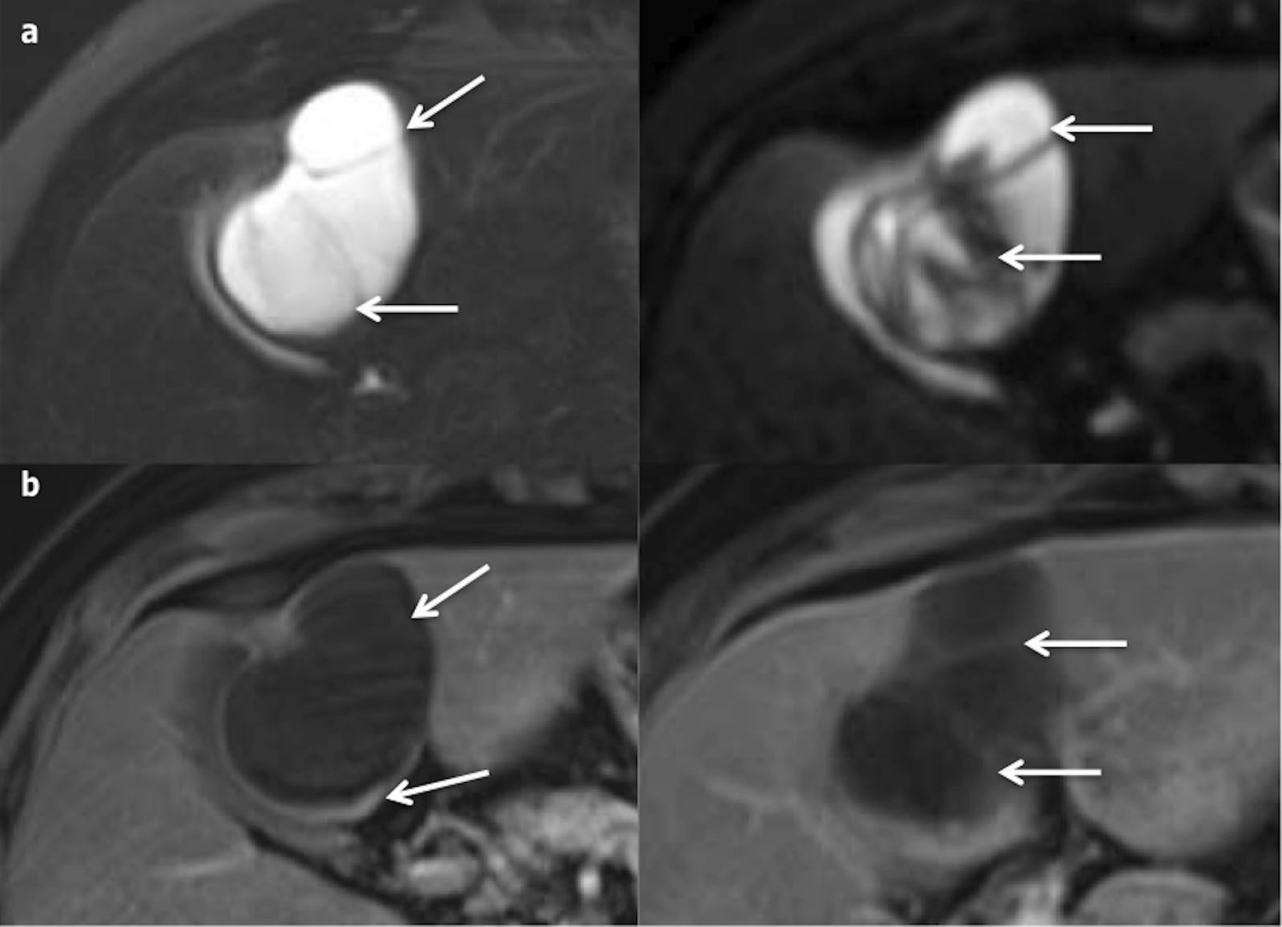
Typical non-invasive mucinous cystic neoplasm of the liver in a 59-year-old female. **a** Axial T2-weighted magnetic resonance imaging shows a solitary hyperintense cystic lesion, with hypointense septa (arrows). **b** Axial T1-weighted magnetic resonance imaging on portal venous phase depicts the enhancement of the capsule and septa (arrows)

On MRI, the signal intensity of the lesion may vary depending on the fluid content within each stall (e.g. protein rich), which is very specific [40]. A fluid–fluid level can be seen when there is haemorrhagic content. The capsule and the septa are enhanced with contrast administration (Fig. 19).

**Fig. 20:**
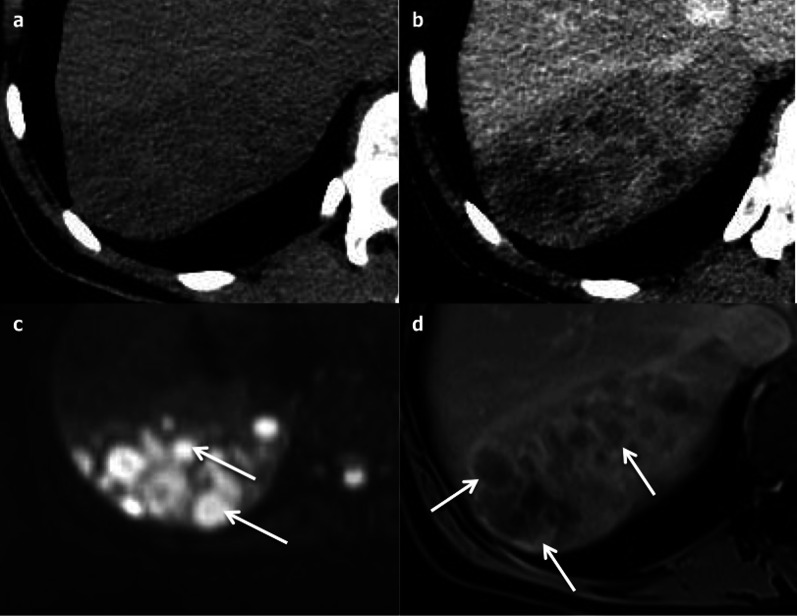
Invasive mucinous cystic neoplasm of the liver in a 50-year-old female. **a** Axial non-enhanced computed tomography shows a hypoattenuating lesion with a thick heterogeneous enhancing wall on portal venous phase (**b**), better seen on T1-weighted magnetic resonance imaging on portal venous phase (**d**) (arrows). Note the presence of cystic portions on axial T2-weighted sequence (**c**), in very intense hypersignal T2 (arrows)

Haemorrhagic fluid, solid mural nodule and calcification are most commonly associated with invasive lesions, mural nodules in particular being highly indicative of malignancy [4] (Fig. 20).

It is difficult to carry out a differential diagnosis with mucinous metastases. Of course, the existence of older imaging is crucial, and the context and history of primary mucinous tumour are important elements to consider. The appearance and evolution under treatment are part of the diagnosis in this case. The presence of septa and mural nodule is atypical for mucinous metastases.

MCN should not be confused with an abscess. Unlike the abscess, the solid part of the lesion and the parietal thickening are asymmetric, with thin-walled and thickened parts. The fluid content of the cystic portion is pure fluid (frank hyperintensity on T2-weighted MRI), and no peripheral oedema is observed (hypoattenuated on CT, hyperintense on MRI).

### Intraductal papillary neoplasm of the bile duct

#### Aetiopathogenesis

Intraductal papillary neoplasm of the bile duct (IPNB) is a rare premalignant neoplasm of the bile duct (10% of all bile duct tumours [41]). However, 40% can contain malignant component [41] and the risk of malignant transformation is high. Lesions must be treated by surgical resection whenever possible [41–43].

#### Imaging

**Fig. 21:**
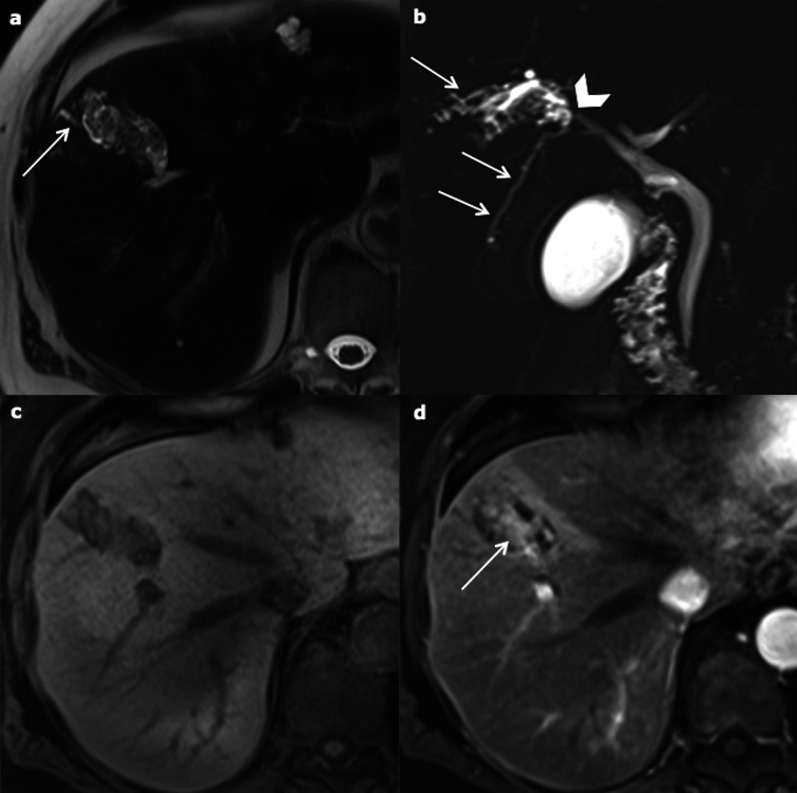
Intraductal papillary neoplasm of the bile duct in a 67-year-old female. **a** Axial T2-weighted magnetic resonance imaging displays intraductal heterogeneous cystic and tissular mass (star) with dilated bile duct in contact (arrows). The cystic part of the mass is better seen at magnetic resonance cholangiopancreatography (**b**), and so are the upstream biliary dilatation (arrows) and the connection to the biliary tree (arrowhead). **c** and **d** Axial T1-weighted non-enhanced magnetic resonance imaging on arterial phase shows a highly enhanced tissular part

The appearance of the lesions depends on the quantity of mucin produced by the tumours [44]. They mainly appear as an intraluminal cystic mass within the bile duct, with upstream duct dilatation (Fig. 21). If there is an increase in the production of mucin, lesions can appear only as focal or diffuse bile duct dilatations, without any visible solid part. The aneurysmal dilatation of a branch of the biliary tree is considered a characteristic sign of IPNB by some authors [45]. An obvious intracystic solid mass is suggestive of a malignant transformation [44]. An intra-luminal mass may appear hypo- or hyperechoic on US, and highly enhanced on CEUS, CT and MRI during the arterial phase (Fig. 21). CEUS is very sensitive for the detection of cystic walls or nodule enhancement [42]. In contrast to intraductal cholangiocarcinoma, enhancement does not increase in the portal and delayed phases [44]. Diffusion may be restricted, but it is not indistinguishable from that of cholangiocarcinoma.

### Secondary cystic lesion: cystic metastasis

#### Aetiopathogenesis

Cystic metastases occur less frequently than solid metastases and purely cystic metastatic tumours without prior treatment are extremely rare. The cystic part of the metastases can be due to spontaneous necrosis, especially in the case of hyperenhancing metastases (neuroendocrine tumours, malignant melanomas, sarcomas or gastrointestinal stromal tumours (GIST)), or it can arise secondary to a systemic or locoregional treatment [3]. There are exceptional cases of cystic liver metastases from squamous cell carcinoma [46]. Mucin production (mucinous colorectal or ovarian adenocarcinoma) can also lead to cystic features.

#### Imaging

**Fig. 22:**
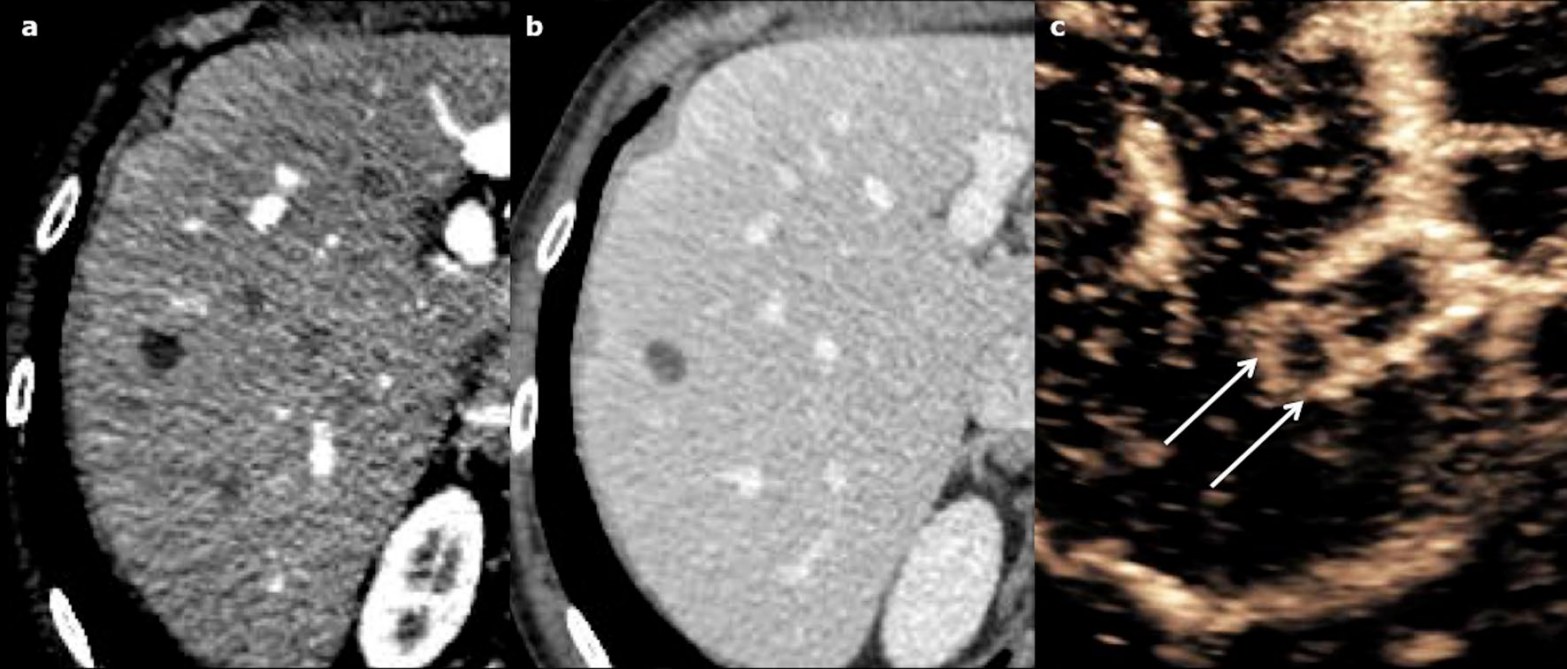
Cystic metastasis from malignant melanoma in a 72-year-old female. **a**, **b** Axial computed tomography on arterial and portal venous phases shows unique hypoattenuating (20 Hounsfield Units) nodule of the right liver lobe, too small to be characterised. **c** Contrast-enhanced ultrasonography displays an enhanced wall as a “rim” (arrows)

**Fig. 23:**
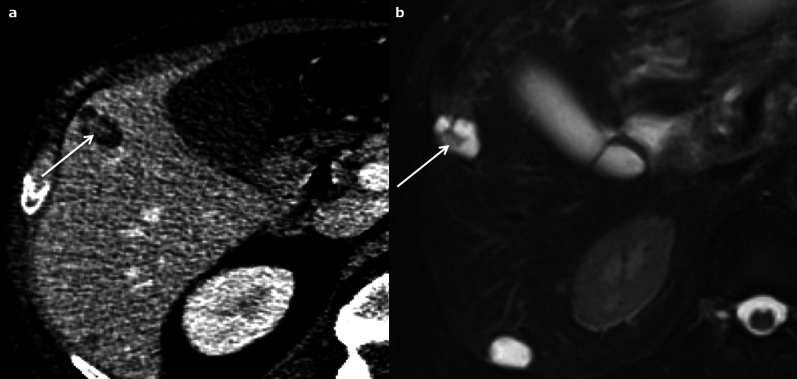
Metastatic pancreatic neuroendocrine tumour in a 60-year-old male. **a** Axial computed tomography on portal venous phase shows a lobulated hypodense cystic nodule in segment V with enhanced mural nodule and septa (arrow), very hyperintense on T2-weighted magnetic resonance imaging (**b**). A second cystic subcapsular nodule is visible in segment VI

**Fig. 24:**
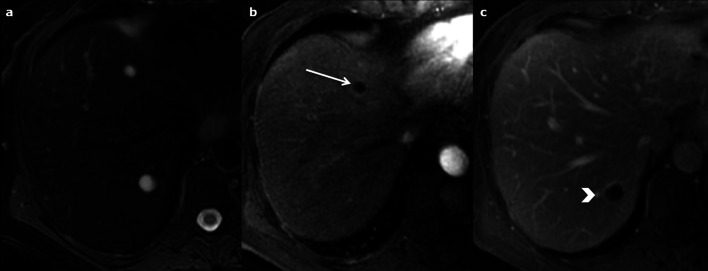
Pure cystic metastasis of a neuroendocrine tumour in a 69-year-old female. The hyperintensity on axial T2-weighted magnetic resonance imaging (**a**) is marked and there is no enhancement on arterial (arrow) or portal venous (arrow-head) phase (**b**, **c**)

**Fig. 25:**
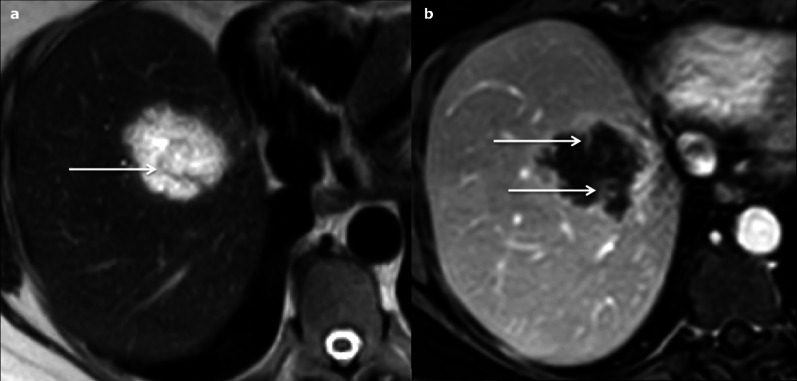
Cystic metastasis of a mucinous tumour of the rectum in a 72-year-old male. The hyperintensity on axial T2-weighted magnetic resonance imaging **a** is marked but the multiple tiny septa (arrows) incompletely enhanced on portal venous phase (**b**) are sufficient to reach a conclusive diagnosis in this the context

They can be multilocular or unilocular, and present thick walls, irregular contours or mural nodules (Figs. 22, 23, 24, 25). Irregular thick septa are possible (Fig. 25). Since the fluid component results from mucin, necrosis or haemorrhage [3], the attenuation on CT and the signal on MRI are different from pure fluid. US may be useful to distinguish truly fluid cystic masses from cystic metastases, especially when they are small. CEUS can reveal fine peripheral arterial enhancement with early washout (Fig. 22). The clinical setting has to be considered.

## Infectious cystic lesions

### Pyogenic abscess

#### Aetiopathogenesis

Frequent organisms of pyogenic abscesses are *Escherichia coli*, *Klebsiella pneumoniae*, *Enterococcus*, and *Streptococcus* [47]. Hyper-virulent strains of *Klebsiella pneumoniae* have been responsible for severe emerging diseases for the past two decades, particularly in Southeast Asia. Some of them are responsible for bacteremia combined with multiple abscesses (monomicrobial liver abscesses, endophthalmitis, brain abscesses) [48].

The differential diagnosis of pyogenic abscess is the amoebaean abscess.

#### Imaging

**Fig. 26:**
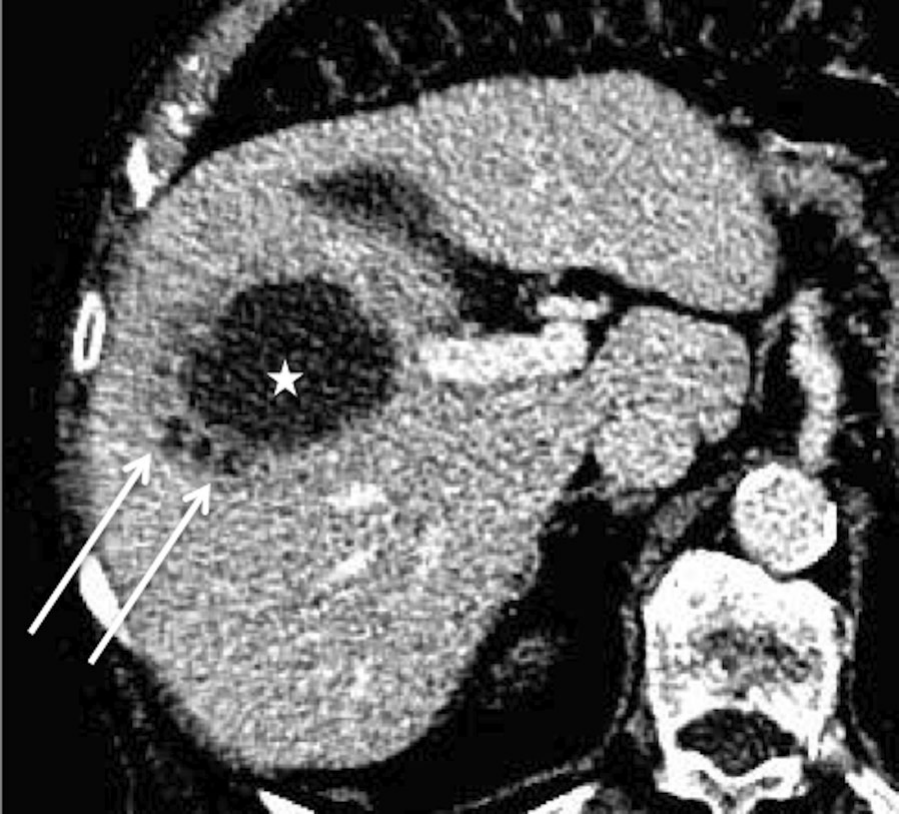
*Escherichia Coli* abscess in an 89-year-old male. Axial computed tomography on portal venous phase shows three small hypoattenuating areas (arrows) forming the “cluster sign” and a large central hypoattenuating cavity (star)

**Fig. 27:**
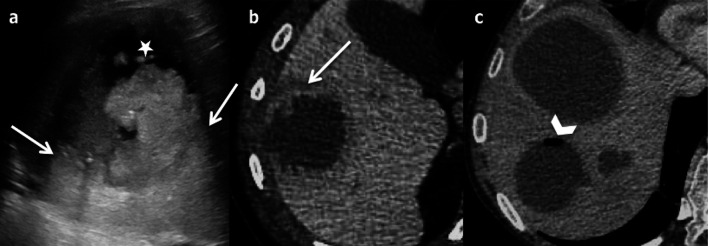
Hepatic pyogenic abscesses in a 49-year-old male. **a** Ultrasonography shows a large heterogeneous hypoechoic lesion, with anechoic parts (star) and increased through transmission (arrows) confirming its cystic nature. **b** and **c** Axial computed tomography on portal venous phase displays multiple abscesses appearing as cystic masses, with a specific sign: the double target (arrows). Note the bubble of gas in one of them (arrow-head)

Abscesses are rarely purely cystic and their appearance varies according to the pathologic stage. Before they turn into cystic abscesses, they display a pre-suppurative phase. Then, they appear as multiple cystic nodules (honeycomb pattern) which coalesce in a unique large irregularly shaped cystic cavity (cluster sign), surrounded by hypoattenuating inflammatory parenchyma (Fig. 26). The cluster sign is a feature indicative of pyogenic abscess [47]. The cystic central part appears on US as a large heterogeneous cystic mass, with mixed anechoic, hypoechoic and hyperechoic parts (Fig. 27). On CT and MRI, a rim and septa enhancement has been reported and the “double target” characteristic sign may be observed. This sign is a hypoattenuating central area of pus surrounded by an inner hyperattenuating ring (granulation tissue) and an outer hypoattenuating zone (inflammatory oedema) [3]. Gas may be present (Fig. 27). The central restriction of diffusion due to pus may be seen in larger abscesses.

The appearance of abscesses can vary due to their pathogenesis: while cholangitic abscesses are mostly multiple small cystic nodules, abscesses by hematogenous dissemination are mostly larger and less numerous, and abscesses by contiguous spread are commonly solitary [49].

The thrombotic occlusion of the portal vein or of its branches, or the hepatic veins, is an additional feature supporting the diagnosis of pyogenic abscess [50].

### Hydatid cyst

#### Aetiopathogenesis

**Fig. 28:**
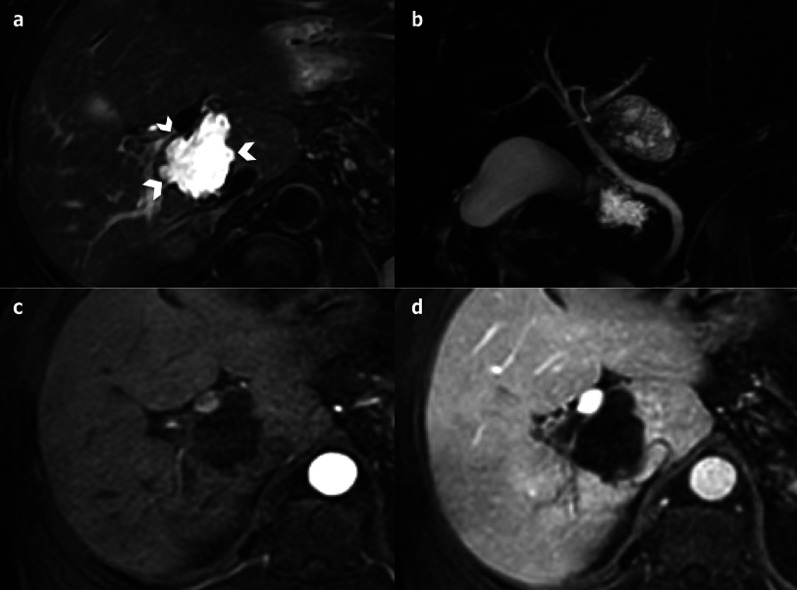
Complicated hydatid cyst in a 50-year-old female. Imaging displays a cyst very close to the biliary tract with lobulated margins, showing a loss of intralesional pressure, most probably resulting from a complication of the biliary connection (not found on magnetic resonance cholangiopancreatography). **a** Axial T2-weighted magnetic resonance imaging. **b** Coronal 3D magnetic resonance cholangiopancreatography. **c** and **d** Axial T1-fat-sat-weighted imaging without and with contrast on portal venous phase

Hydatid cysts are caused by *Echinococcus granulosis* infection [3]. They consist of three layers: the outer pericyst, which corresponds to compressed and fibrosed liver tissue; the endocyst, an inner germinal layer; and the ectocyst, a thin, translucent interleaved membrane [51]. The cysts are mainly asymptomatic, but they can become complicated (biliary fistula, compression, rupture) (Fig. 28) [51].

#### Imaging

Their characteristic appearance varies, depending on the stage of the disease. On US, both the World Health Organization (WHO) and the Gharbi classification systems are used.

**Fig. 29:**
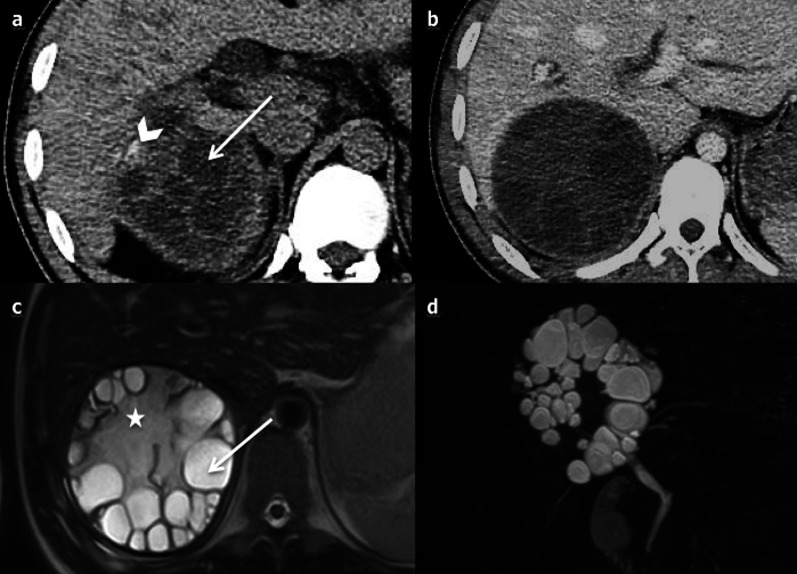
cyst echinococcosis 2, in a 50-year-old male. **a** Axial non-enhanced computed tomography shows a heterogeneous mass with a cystic part (arrow) and calcification of the wall (arrowhead). **b** On axial computed tomography on portal venous phase no enhancement of the cystic component is observed. **c** The cystic part and the differentiation between the daughter (arrow) and the mother (star) cysts are better evaluated on axial T2-weighted magnetic resonance imaging. **d** Coronal 3D magnetic resonance cholangiopancreatography excludes biliary fistula from the possible diagnosis

The WHO classification system contains seven categories, which depend on the stage of the disease. The first category is called cystic lesion (CL). These are unilocular anechoic lesions, which appear as simple hepatic cysts. CE1 (cystic echinococcosis 1) corresponds to an active stage: it appears as a uniform anechoic cyst with internal echoes (“hydatid sand”) on US, which may only be seen after patient repositioning. A peripheral hypointense capsule is visible on MRI. CE2 (active stage) (Fig. 29) is as a well-defined multicystic mass with septa. Half of the cases demonstrate calcification of the cyst wall [51, 52], which can be better seen on CT. It displays a multivesicular “honeycomb” appearance due to daughter cysts surrounding a larger “mother” cyst. On CT, the attenuation of the mother cyst is usually higher than that of the daughter cysts due to debris (hydatid sand and detached cysts wall); the signal intensity of the mother cyst is, respectively, higher and lower on T1- and T2-weighted MRI (Fig. 29). T2-weighted MRI sequences are more sensitive than CT to detect and characterise the cystic mass as a typical wheel-spoke pattern (Fig. 29). T2 images show septa and low signal intensity due to the presence of calcifications and to the fibrotic component. They have a different signal on T1-weighed images due to the cyst fluid, which varies according to the amount of proteinaceous debris [3]. Diffusion is not restricted [52]. CE3 is a transitional stage. CE3A is a unique cyst with detached laminated membranes described as the “water lily sign”[51–53]. CE3B shows daughter cysts within a solid matrix. CE4 corresponds to an inactive stage. There are no more daughter cysts. It produces both hypoechoic and hyperechoic signal, whose pattern resembles a ball of wool [53]. (inactive/degenerative stage), is partially or completely calcified.

### Hepatic alveolar echinococcosis

#### Aetiopathogenesis

This is a rare parasitic infection due to *Echinococcus multilocularis*. In contrast to the hydatid cyst, a fibrous capsule does not usually demarcate the outer margin. It is a chronic and latent disease, which is usually detected incidentally [52]. When it is symptomatic, it causes compression symptoms. The treatment is medical and surgical. The prognosis is usually poor, sometimes leading to liver transplantation.

#### Imaging

Hepatic alveolar echinococcosis (HEA) lesions present irregular margins, with a mixed component of solid and cystic parts. Calcifications frequently occur. The lack of enhancement is very helpful to reach a diagnosis.

**Fig. 30:**
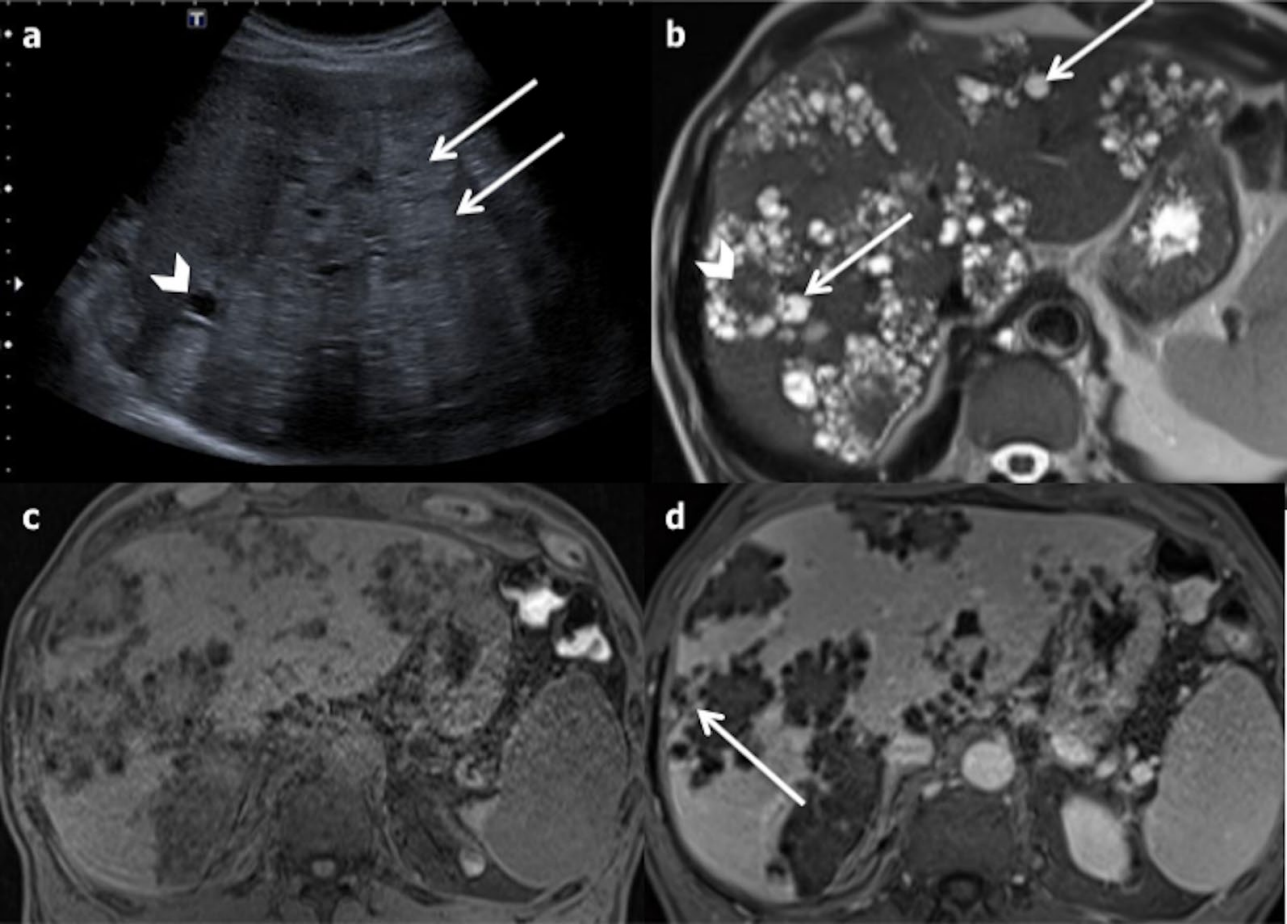
Hepatic alveolar echinococcosis in a 40-year-old male. **a** Ultrasonography shows multiple round hyperechoic nodules (arrows) with cystic anechoic parts (arrowhead). **b** On Axial T2-weighted magnetic resonance imaging, the lesions appear as multivesicular cystic masses (arrows) with a fibrous component (arrowhead). **c** and **d** On axial non-enhanced T1-fat-sat-weighted magnetic resonance imaging, the fibrous part is enhancing on late phase (arrow)

On US, HEA lesions appear as a large heterogeneous mass, with areas of hyper- and hypoechogenicity (Fig. 30), hyperechoic spots (corresponding to calcifications), and a central cystic anechoic cavity (due to necrosis) [52]. More rarely they appear as multiple hyperechoic nodules with calcification and a cystic part. CT is helpful to identify the calcification. The lesions, which are hypoattenuating, heterogeneous, with irregular margins and calcification, may attract the surrounding parenchyma and distort the hepatic capsule. Usually, no contrast enhancement is observed. In the late phase, the fibrotic component may display mild enhancement. The use of MRI prior to surgery is crucial, because it helps to assess the diagnosis, demonstrating the multivesicular structure of the lesion, and allowing vascular or biliary tree involvement to be assessed [52]. The signal intensity of the cystic part ranges from low to intermediate on T1-weighted images and is high on T2-weighted images; the fibrous part demonstrates low signal intensity both on the T1- and the T2-weighted images (Fig. 30).

Therapeutic efficacy in HAE is essentially evaluated on the basis of repeated 18-F-FDG PET CT [54].

## Conclusion

Cystic liver lesions encompass a wide and varied spectrum of features. To characterise them, a particular attention should be paid to wall thickness, to the presence of enhanced septa or solid components, to the fluid signal on MRI and to the clinical context. Since cystic liver lesions may be malignant, all non-SHCs should be investigated to make a conclusive diagnosis.

## Abbreviations

18-F-FDG PET: 18-Fluorine-fluorodeoxyglucose positron emission tomography
ADPKD: Autosomal dominant polycystic kidney disease
ADPLD: Autosomal dominant polycystic liver disease
BH: Bile duct hamartomas
CA19-9: Carbohydrate antigen 19-9
CD: Caroli disease
CE: Cystic echinococcosis
CE1: Cystic echinococcosis 1
CE2: Cystic echinococcosis 2
CE3: Cystic echinococcosis 3
CE4: Cystic echinococcosis 4
CE5: Cystic echinococcosis 5
CEA: Carcinoembryonic antigen
CEUS: Contrast-enhanced ultrasonography
CHF: Congenital hepatic fibrosis
CHFC: Ciliated hepatic foregut duplication cysts
CL: Cystic lesion
CS: Caroli syndrome
CT: Computed tomography
GIST: Gastrointestinal stromal tumour
HC: Hepatic cysts
HEA: Hepatic alveolar echinococcosis
HLM: Hepatic lymphatic malformation
HU: Hounsfield units
IPNB: Intraductal papillary neoplasms of the bile duct
MCN-L: Mucinous cystic neoplasms of the liver
MRCP: Magnetic resonance cholangiopancreatography
MRI: Magnetic resonance imaging
PLD: Polycystic liver disease
SHC: Simple hepatic cysts
TAG-72: Tumour-associated glycoprotein 72
US: Ultrasonography
WHO: World health organization
